# Nodule organogenesis in *Medicago truncatula* requires local stage-specific auxin biosynthesis and transport

**DOI:** 10.1093/plphys/kiaf133

**Published:** 2025-04-04

**Authors:** Ting Ting Xiao, Sophia Müller, Defeng Shen, Jieyu Liu, Kelvin Adema, Amber van Seters, Henk Franssen, Ton Bisseling, Olga Kulikova, Wouter Kohlen

**Affiliations:** Department of Plant Sciences, Cluster of Plant Developmental Biology, Laboratory of Molecular Biology, Wageningen University, Droevendaalsesteeg 1, Wageningen 6708 PB, The Netherlands; Department of Plant Sciences, Cluster of Plant Developmental Biology, Laboratory of Cell and Developmental Biology, Wageningen University, Droevendaalsesteeg 1, Wageningen 6708 PB, The Netherlands; Department of Plant Sciences, Cluster of Plant Developmental Biology, Laboratory of Molecular Biology, Wageningen University, Droevendaalsesteeg 1, Wageningen 6708 PB, The Netherlands; Department of Plant Sciences, Cluster of Plant Developmental Biology, Laboratory of Molecular Biology, Wageningen University, Droevendaalsesteeg 1, Wageningen 6708 PB, The Netherlands; Department of Plant Sciences, Cluster of Plant Developmental Biology, Laboratory of Cell and Developmental Biology, Wageningen University, Droevendaalsesteeg 1, Wageningen 6708 PB, The Netherlands; Department of Plant Sciences, Cluster of Plant Developmental Biology, Laboratory of Cell and Developmental Biology, Wageningen University, Droevendaalsesteeg 1, Wageningen 6708 PB, The Netherlands; Department of Plant Sciences, Cluster of Plant Developmental Biology, Laboratory of Molecular Biology, Wageningen University, Droevendaalsesteeg 1, Wageningen 6708 PB, The Netherlands; Department of Plant Sciences, Cluster of Plant Developmental Biology, Laboratory of Molecular Biology, Wageningen University, Droevendaalsesteeg 1, Wageningen 6708 PB, The Netherlands; Department of Plant Sciences, Cluster of Plant Developmental Biology, Laboratory of Molecular Biology, Wageningen University, Droevendaalsesteeg 1, Wageningen 6708 PB, The Netherlands; Department of Plant Sciences, Cluster of Plant Developmental Biology, Laboratory of Molecular Biology, Wageningen University, Droevendaalsesteeg 1, Wageningen 6708 PB, The Netherlands; Department of Plant Sciences, Cluster of Plant Developmental Biology, Laboratory of Cell and Developmental Biology, Wageningen University, Droevendaalsesteeg 1, Wageningen 6708 PB, The Netherlands

## Abstract

The importance of auxin in plant organ development, including root nodule formation, is well known. The spatiotemporal distribution pattern of auxin during nodule development has been illustrated using auxin reporter constructs. However, our understanding of how this pattern is established and maintained remains elusive. Here, we studied how the auxin gradient is associated with the spatiotemporal expression patterns of known auxin biosynthesis and transport genes at different stages of nodule development in Medicago (*Medicago truncatula*). In addition, we examined the Medicago PIN-FORMED10 (*Mt*PIN10) expression pattern and polar positioning on the cell membrane during nodule primordium development to investigate auxin flux. RNA interference and the application of auxin biosynthesis inhibitors were used to demonstrate the importance of auxin biosynthesis and transport at the initial stages of nodulation. Our results show that upon rhizobium inoculation before the first cell divisions, a specific subset of Medicago *YUCCA* (*MtYUC*) and *MtPIN* genes, as well as Medicago *LIKE AUXIN RESISTANT2* (*MtLAX2*), are expressed in the pericycle and contribute to the creation of an auxin maximum. Overall, we demonstrate that the dynamic spatiotemporal expression of both *MtYUC* and *MtPIN* genes results in specific auxin outputs during the different stages of nodule primordia and nodule meristem formation.

## Teaser

Dynamic spatiotemporal expression of auxin biosynthesis and auxin transport carrier genes results in specific auxin outputs during different stages of nodule primordia and nodule meristem formation.

## Introduction

In legumes, rhizobium can establish a symbiotic relationship leading to the formation of nitrogen-fixing root nodules, involving 2 different developmental programs: infection thread initiation in the epidermis and nodule primordium formation in the inner root cell layers. Nodule formation is initiated by lipo-chitooligosaccharides, called Nod factors, secreted by rhizobia (reviewed by Dénarié et al. 1996; Geurts and Bisseling 2002). In Medicago (*Medicago truncatula*), rhizobia enter the root via root hairs, where tubular structures, called infection threads, are formed (reviewed by Brewin 2004; Gage 2004). Concomitantly, differentiated root cells, including pericycle and cortical cells, are mitotically reactivated in an organized manner by which the Medicago nodule primordium is formed (reviewed by Oldroyd and Downie 2006; Xiao et al. 2014). The infection thread that contains rhizobia grows toward the primordium, and bacteria are released in cells derived from the inner cortex. A meristem which facilitates further growth is formed at the apex of the nodule primordium.

In general, 2 types of nodules can be distinguished based on the lifespan of the meristem. In determinate nodules (e.g. lotus, *Lotus japonicus*), the meristem is active for a short time, while in indeterminate nodules (e.g. Medicago), a persistent meristem is formed (reviewed by Hirsch 1992; Sprent and James 2007). These plants also differ in the location of the first cell divisions initiated upon rhizobial inoculation. Indeterminate nodules are initiated in the pericycle and the inner cortical cells and determinate nodules start in the outer and middle cortical cells (reviewed by Hirsch 1992). In each nodule type, the initiation of cell divisions is associated with auxin accumulation (Mathesius et al. 1998b; Pacios-Bras et al. 2003; Wasson et al. 2006; Mathesius 2008; Zhang et al. 2009; Ng et al. 2015; Herrbach et al. 2017; Ng and Mathesius 2018).

Irrespective of the nodule type formed, auxin is involved in cell cycle control, vascular tissue differentiation, and rhizobial infection during nodule development (reviewed by Kohlen et al. 2018; Lin et al. 2020). Auxin is mainly synthetized in the aerial part of the plant and can be transported toward the root apex both passively along the phloem and actively by auxin transporters (Swarup et al. 2001; Vanneste and Friml 2009; Swarup and Péret 2012; Swarup and Bennett 2014). Several studies have shown that inhibitors that block this acropetal auxin transport induce the formation of pseudonodules by creating a local auxin maximum (Allen et al. 1953; Hirsch et al. 1989; Scheres et al. 1992; Schnabel and Frugoli 2004; Subramanian et al. 2006; Wasson et al. 2006; Zhang et al. 2009; Rightmyer and Long 2011; Shen et al. 2015; Sańko-Sawczenko et al. 2016). An inhibition of auxin transport toward the root apex at the very start of nodule initiation was observed during the formation of indeterminate, but not determinate, nodules. On the contrary, at the initiation of determinate nodules, an increase in acropetal auxin transport was observed (Allen et al. 1953; Hirsch et al. 1989; Rubery and Jacobs 1990; Mathesius et al. 1998b; Boot et al. 1999; Pacios-Bras et al. 2003; Prayitno et al. 2006; Subramanian et al. 2006). Polar auxin transport is mediated by efflux carriers, among them the best studied are PIN-FORMED (PINs) and auxin influx carriers AUXIN RESISTANT1 (AUX1) and LIKE-AUXIN RESISTANT1 (LAX) (Petrásek et al. 2006; Swarup and Bhosale 2019). The formation of a PIN-dependent local auxin gradient is a central part of several developmental processes in plants (Benková et al. 2003; Wisniewska et al. 2006). In Medicago, 12 *PIN* genes were previously identified (Schnabel and Frugoli 2004; Shen et al. 2015; Sańko-Sawczenko et al. 2016; Kohlen et al. 2018). In experiments where Nod factors or *Sinorhizobium meliloti* was applied to Medicago (A17), it was shown that the earliest induced *PINs* in Medicago are *MtPIN2*, *MtPIN4*, and *MtPIN10* (Plet et al. 2011; van Zeijl et al. 2015; Schiessl et al. 2019). The expression of these *MtPINs* during nodule initiation and development and reported phenotypes of *MtPIN*-*RNAi* plants in Medicago indicate their importance in the creation of the auxin landscape during nodule development (Huo et al. 2006; Shen et al. 2015; Sańko-Sawczenko et al. 2016; Schiessl et al. 2019).

In addition to PINs, AUX1/LAX proteins function as auxin influx carriers. There are 5 members of the AUX1/LAX family in Medicago (Schnabel and Frugoli 2004). The expression of *MtLAX2*, a homolog of *AtAUX1*, is highly induced during the initiation of nodule primordia (Roy et al. 2017; Schiessl et al. 2019). Moreover, 2 identified Medicago *Tnt1* insertion mutants affected in this gene, *Mtlax2-1* and *Mtlax2-2*, developed fewer nodules and lateral roots, suggesting the same requirements for auxin influx activity for development of both lateral organs (de Billy et al. 2001; Schnabel and Frugoli 2004; Swarup and Péret 2012; Roy et al. 2017). In line with the putative function of the *Mt*LAX2 protein, both mutants displayed decreased activity of the synthetic auxin response promoter DR5 fused to β-glucuronidase (*DR5::GUS*) associated with rhizobial infection (Roy et al. 2017).

Auxin maxima are not only established as the result of polar auxin transport but also as a consequence of local auxin biosynthesis (reviewed by Zhao 2018; Cao *et al.* 2019). Although there are several pathways that lead to the production of auxin, the TRYPTOPHAN AMINOTRANSFERASE (TAA)/YUCCA (YUC) pathway is reported to be the primary biosynthesis route for endogenous auxin (reviewed by Zhao 2010, 2018). The TAA/YUC pathway is a 2-step pathway in which TAA first converts tryptophane into indole-3-pyruvic acid (IPyA). Next, YUC, a flavin-containing monooxygenase, catalyzes the rate-limiting step in biosynthesis of indole-3-acetic acid (IAA), the main natural auxin, from IPyA (Mashiguchi et al. 2011; Stepanova et al. 2011; Won et al. 2011). In Medicago, 4 *YUC* genes were previously reported to be upregulated during nodule primordium formation: *MtYUC1*, *MtYUC*2, *MtYUC8*, and *MtYUC9*, and their expression is (in)directly regulated by the root nodule symbiosis-specific transcription factor NODULE INCEPTION (NIN) (Liu et al. 2019; Schiessl et al. 2019).

Overall, during nodule initiation, it is likely that defined auxin concentrations are needed to induce and guide nodule organogenesis. However, how the auxin-related gene network contributes to the establishment of such concentrations remains unknown. To unravel the complexities of these highly interconnected molecular physiological networks, we have employed a combination of techniques, including *DR5::GUS*-based visualization of auxin output and the spatiotemporal expression profiling of genes associated with auxin biosynthesis and transport, known to be expressed during the formation of a nodule primordium. We observed that the accumulation of auxin precedes cell divisions and, in subsequent stages of Medicago primordium development, remains closely associated with mitotic reactivation. Our investigations have revealed both distinct and overlapping spatiotemporal expression patterns for *MtYUC* and *MtPIN* genes. Additionally, we have pinpointed the localization of *Mt*PIN10 at the inception of nodule initiation and during subsequent developmental stages. Furthermore, through the utilization of *RNAi* and pharmacological interventions, we have validated that in addition to auxin transport, local auxin biosynthesis is an indispensable contributor to the initiation and development of root nodules in Medicago.

## Results

### Defining the auxin response pattern during nodule initiation

To create a frame of reference for spatiotemporal analyses of genes involved in auxin patterning, we first determined the dynamics of the auxin gradient during nodule primordium initiation and development as previously described (Xiao et al. 2014). For this, we created a stable transgenic Medicago R108 line containing the synthetic auxin response promoter DR5 fused to β-glucuronidase (*DR5::GUS*).

**Figure 1.**
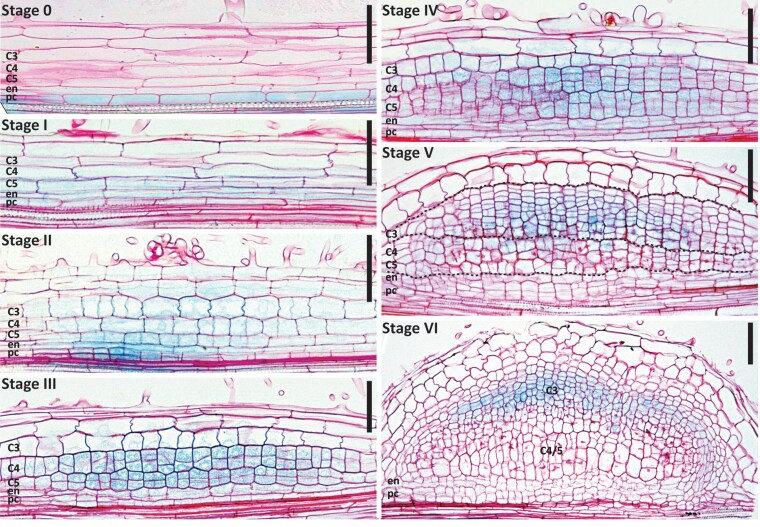
*DR5::GUS* expression dynamics during the different stages of nodule primordium development in Medicago (cv R108). Longitudinal plastic sections (10 mm) of spot-inoculated root segments counterstained with ruthenium red. Stage 0 (between 12 and 24 hpi), *DR5* is activated in pericycle cells preceding their division. Stage I (∼24 hpi), first anticlinal pericycle divisions have occurred; the *DR5::GUS* signal extends to the cortical cell layers prior to their activation. Stage II (∼30 hpi), anticlinal divisions extend to cortex layers 3, 4, and 5; broad DR5 activity is detected in the entire nodule primordia. Stage III (∼48 hpi), periclinal cell divisions in cortex layers 4 and 5; *DR5* is highly expressed in actively dividing cortical cells of these layers. Stage IV (∼72 hpi), periclinal cell divisions in cortex layer 3; DR5 signal extends to the third cortical cell layer. Stage V (between 72 and 96 hpi), cells derived from the C3 layer actively divide to form the future nodule meristem; DR5 activity is highest in these cells. Pericycle, endodermis, and cortex layers 4 and 5 have stopped dividing and DR5 activity is no longer detected in the center of these cell layers. Stage VI (>96 hpi), vasculature bundles are formed, nodule meristem becomes active, and DR5 activity is restricted to the nodule vasculature, nodule meristem, and infected cells directly adjacent to it. For each time point, 10 to 15 spot-inoculated root segments were sectioned. Most representative images for each stage were selected to be shown. C3-C5, cortical cell layers; en, endodermis; pc, pericycle; scale bars 75 *μ*m.

Plants were grown on Fähraeus (Fä) plates containing 1 *µ*M aminoethoxyvinylglycine (AVG) as previously described (Fähraeus 1957; Xiao et al. 2014). Roots of these plants were collected at different time points after spot inoculation with *S. meliloti* 2011 (*Sm2011*) and incubated in GUS substrate buffer. Segments of stained roots were cut, fixed, embedded in plastic, and subsequently sectioned (Fig. 1). The earliest *DR5* activity was observed in pericycle cells prior to periclinal divisions. As at this stage (12 to 24 h postinoculation [hpi]), pericycle cell divisions were not yet visible, we refer to this stage as Stage 0 of nodule initiation. Stage 0 directly precedes the previously described Stage I with detectable pericycle cell divisions (Xiao et al. 2014). Stage 0 can be distinguished from lateral root initiation in the number of pericycle cells activated. Where lateral root initiation sites in Medicago are usually between 2 and 4 pericycle cells wide, at nodule Stage 0, they can vary between 8 and 12 cells (Fig. 1; Supplementary Fig. S1).

Slightly later in Stage I (∼24 hpi), *DR5* activity was observed in dividing pericycle and endodermal cells, and not yet dividing cells of the inner root cortex (C4/5). At Stage II (∼30 hpi), *DR5* activity was maintained in these inner layers and additionally had progressed to the outer cortex layers (C1 to C3) and epidermis. At Stages III (∼48 hpi) and IV (∼72 hpi), *DR5* was highly active in C4/5-derived cells compared to pericycle and endodermis cells. The latter associates with a decrease or completion of cell divisions in these cell layers at those stages.

During Stage V (between 72 and 96 hpi), *DR5* activity in the C4/5-derived cells in the central region of the nodule primordium markedly decreased and was no longer detectable in these cells during Stage VI (96 hpi). In contrast, *DR5* was highly active in C3-derived cells, an activity possibly linked to the establishment of the future nodule meristem from these cells.

In addition, in young nodules, the infected cells directly adjacent to the nodule meristem, as well as cells of the vascular bundles, displayed relatively high *DR5* activity (Supplementary Fig. S2). This pattern is reminiscent of the pattern of *DR5* activity in young Medicago Jemalong A17 (A17) nodules (Demina et al. 2019). Since the majority of experiments that will be described here were conducted in Medicago Jemalong A17 (A17), we compared *DR5* expression patterns between R108 and A17 during early stages of nodule development. To this end, we introduced *DR5::GUS* to A17 by *Rhizobium rhizogenes* (formerly *Agrobacterium rhizogenes*; Young et al. 2001) mediated hairy root transformation. The composite plants were grown in perlite for 7 days and then inoculated with *Sm2011*. At 7 days postinoculation (7 dpi), roots were collected and incubated in GUS substrate buffer. Segments of roots with stained nodule primordia at different stages were cut, fixed, embedded in plastic, and sectioned. A comparison of R108 sections with those representing stages of A17 nodule formation (Supplementary Fig. S3) showed that, despite R108 being stably transformed and A17 transiently transformed, the *DR5* patterns in both ecotypes were very similar.

### Expression patterns of MtYUCs during nodule initiation and development

**Figure 2.**
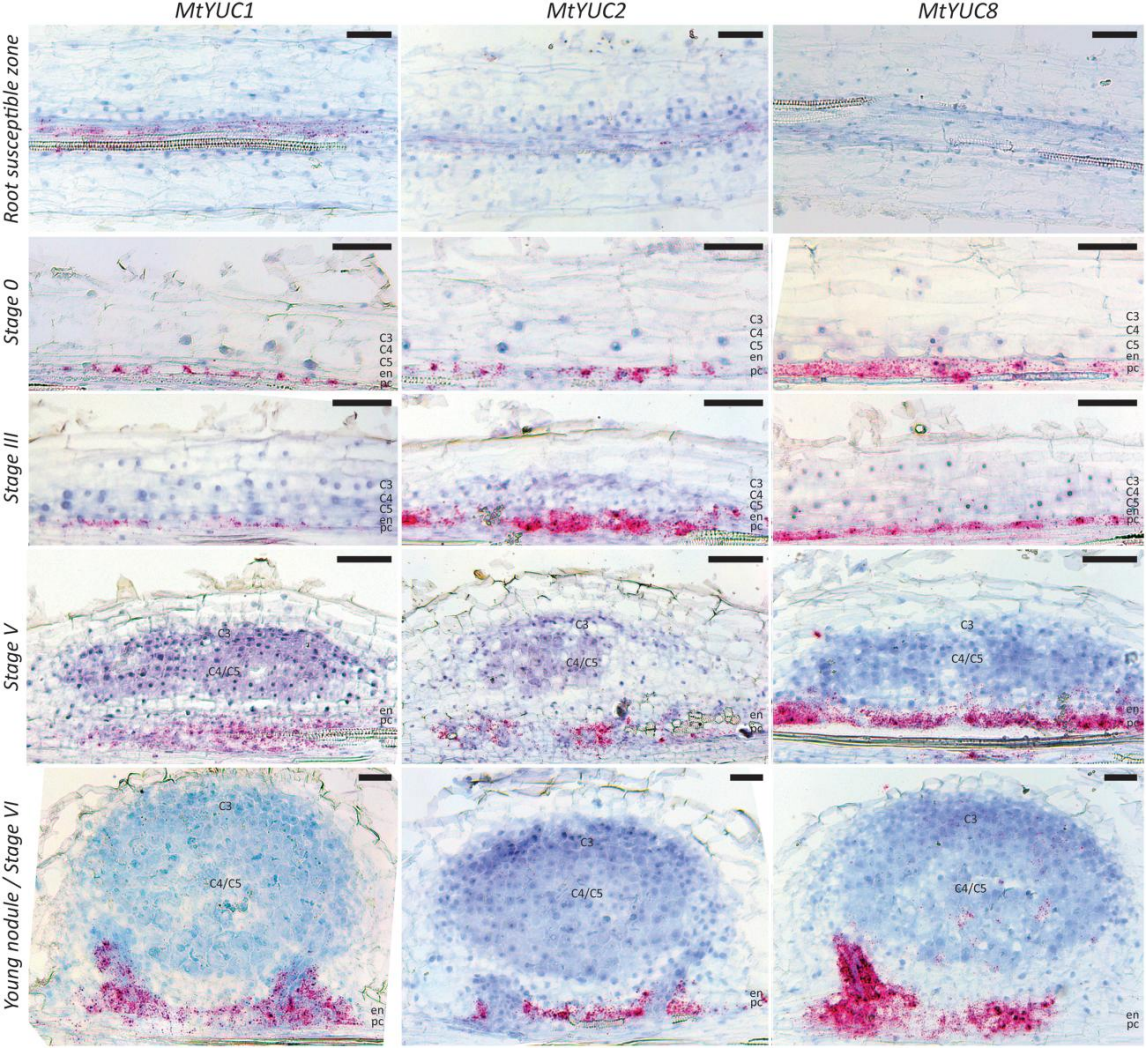
The spatiotemporal expression dynamics of Medicago *YUCCAs* (*MtYUCs*) in the root susceptible zone and during nodule primordium formation. Representative images of RNA in situ hybridizations with *MtYUC1*, *MtYUC2*, or *MtYUC8* probe sets on longitudinal sections of root segments and nodule primordia at different stages of development. Magenta dots are hybridization signals. C3-C5, cortical cell layers; en, endodermis; pc, pericycle; scale bars 75 *μ*m.

*DR5* activity results from auxin gradients established by a dynamic interplay between auxin biosynthesis and transport (reviewed by Zhao 2018; Cao et al. 2019). We first focused on the auxin biosynthesis genes previously reported to be expressed in the root susceptible zone and during nodule primordium formation in Medicago, *MtYUC1*, *2*, *8*, and *9* (van Zeijl et al. 2015; Schiessl et al. 2019). To dissect the role of these *MtYUC* genes in establishing the auxin patterning during nodule development as reflected by *DR5* activity, we first analyzed their spatiotemporal expression patterns in the root tip and susceptible zone. RNA in situ hybridization demonstrated that only *MtYUC1* transcripts were clearly detectable in the vasculature of the root susceptible zone (Fig. 2). All 4 *MtYUC* genes were expressed in the root elongation zone, and the expression of *MtYUC2* and *8* was extended slightly further to the root differentiation zone (Supplementary Fig. S4). In addition, *MtYUC8* and *9* transcripts were observed in the lateral root cap and columella cells of the root tip (Supplementary Fig. S4).

To map the spatiotemporal expression dynamics of these *MtYUC*s onto the *DR5* pattern during nodule primordium organogenesis, we spot-inoculated Medicago roots and performed RNA in situ hybridization on longitudinal root sections accommodating nodule primordium developmental Stages 0 to VI (Fig. 2). For 2 out of 4 *MtYUCs*, *MtYUC1*, *2*, and *8*, a strong hybridization signal was observed in the pericycle at Stage 0. At the Stages III to VI, *MtYUC1*, *2*, and *8* transcripts remained restricted to the pericycle and the forming vasculature. In agreement with previously published data, we did not observe *MtYUC9* transcripts at the early stages of nodule primordia development (Supplementary Fig. S5; Schiessl et al. 2019). From Stage IV onwards, *MtYUC9* transcripts were detectable at the periphery of the nodule primordium. Probably, these expression domains link to cells of the developing nodule vasculature. Across all analyzed stages, *MtYUC9* expression levels were relatively low (Supplementary Fig. S5).

### Functional analysis of MtYUCs during nodule initiation

**Figure 3.**
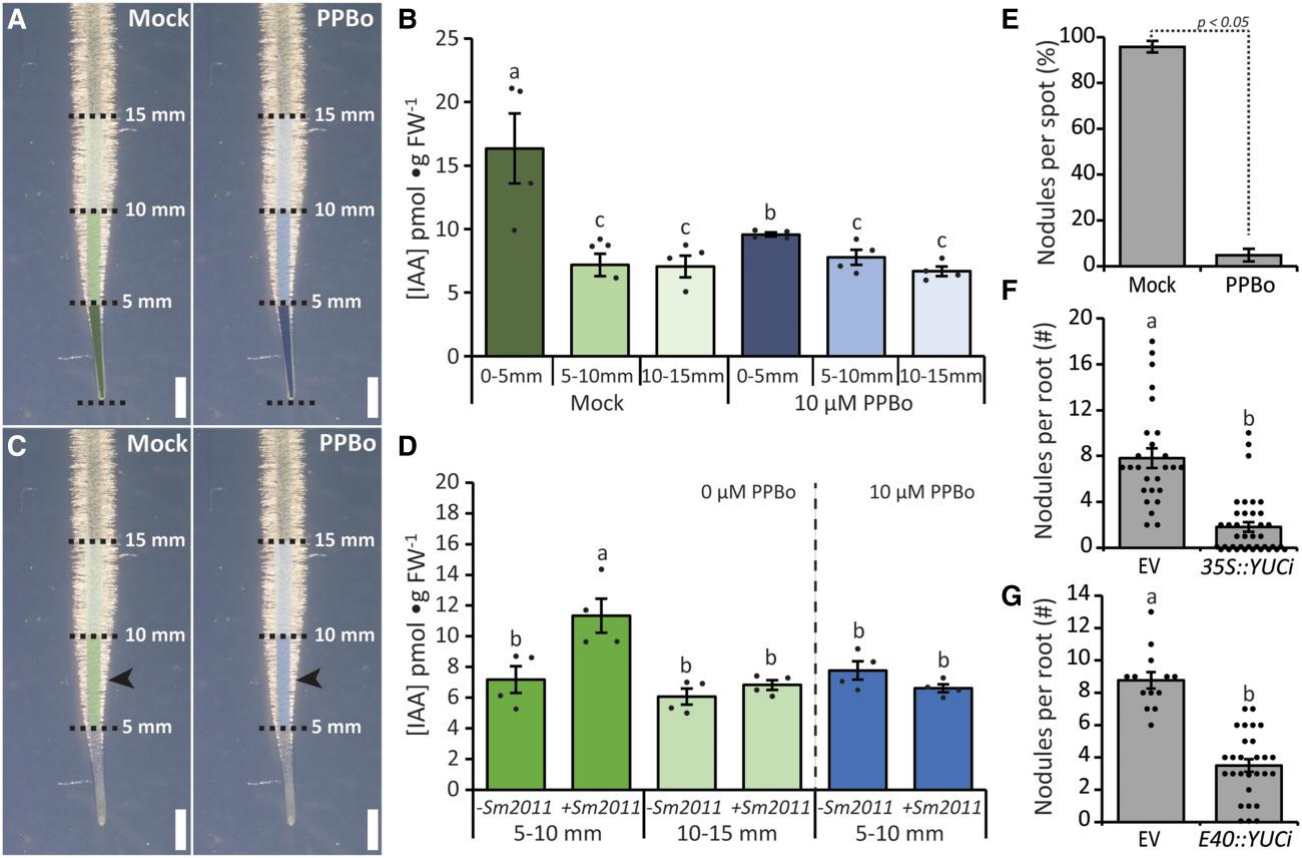
The role of auxin biosynthesis during nodulation. **A** and **B)** The measurement of auxin (IAA) in 3 zones of roots (root tip, susceptible zone, differentiation zone) after 3 h mock or 10 *µ*M 4-PPBo application, bars represent averages, picomole•gram FW^−1^ (pmol•g FW^−1^) ± standard error, dots show individual data points per sample, and different letters indicate significant differences with *P* < 0.05 according to ANOVA and Tukey post hoc test (*n* = 4). **C** and **D)** The effect of 24 h 10 *µ*M PPBo application on rhizobia induced specific auxin (IAA) accumulation in the root susceptible zone, bars represent averages, pmol•g FW^−1^, ±standard error, dots show individual data points per sample, and different letters indicate significant differences with *P* < 0.05 according to ANOVA and Tukey post hoc test (*n* = 4). **A** and **C)** Scale bars 2 mm. **C)** Arrowheads indicate spot application site. **E)** The effect of pharmacological YUCCA inhibition by 10 *µ*M PPBo on the nodulation efficiency of rhizobia spot applications, bars represent combined averages of 4 independent experiments each containing >10 roots, ±standard error, *P* < 0.05 according to Student's *t*-test, total *n* > 40. **F)** Number of nodules formed on *35S_pro_::YUC1/2/8i* (*35S::YUCi*) transgenic roots compared to *EV* control roots, bars represent averages, ±standard error, dots show individual data points per transgenic root, and different letters indicate significant differences with *P* < 0.05 according to ANOVA and Tukey post hoc test (*n* > 25). **G)** Number of nodules formed on *ENOD40_pro_*::*YUC1/2/8i (E40::YUCi)* transgenic roots compared to *EV* control roots, bars represent averages, ±standard error, dots show individual data points per transgenic root, and different letters indicate significant differences with *P* < 0.05 according to ANOVA and Tukey post hoc test (*n* > 13).

Based on the spatiotemporal dynamics of *MtYUC* expressions during nodule initiation, we hypothesized that local auxin biosynthesis in the pericycle is required for nodule initiation. This raises the question whether inhibition or downregulation of *MtYUCs* leads to a reduction in nodulation. To test this, we adopted pharmacological and *RNA* interference approaches (Fig. 3).

It has previously been reported that 10 *μ*M of 4-phenoxyphenylboronic acid (PPBo) is very efficient in blocking YUC enzymatic activity in Arabidopsis (*Arabidopsis thaliana*) (Kakei et al. 2015). To validate that 10 *μ*M PPBo is able to block IAA biosynthesis in our experiments, we first set out to test its efficiency on Medicago roots. We sampled the lower 1.5 cm of uninoculated roots into 3 × 5 mm segments (Fig. 3A) and determined the IAA concentration in these segments (Fig. 3B). This revealed that IAA levels were approximately 2 times higher in the tip of the root compared to the other 2 segments (16.34, 7.18, 7.05 pmol•g^−1^ fresh weight [FW], respectively). The application of 10 *μ*M PPBo for 3 h reduced the level of IAA in the root tip by ∼40%, while not affecting the level of IAA in the 2 other segments (9.57, 7.78, 6.68 pmol•g^−1^ FW respectively; Fig. 3B). The fact that under these conditions *MtYUC* expression is mostly restricted to the root tip (Fig. 2; Supplementary Fig. S4) suggests that 10 *μ*M PPBo was able to block local IAA biosynthesis in the root tip while not affecting the IAA derived from the acropetal auxin transport. Next, we treated roots for 24 h with 10 *μ*M PPBo or mock, and subsequently spot-inoculated these roots with rhizobia in the root susceptible zone (5 to 10 mm). These regions and the 5 mm root segments above (10 to 15 mm) were harvested for IAA quantification at 24 hpi. In line with previous reports (Ng et al. 2015; Schiessl et al. 2019), *Sm2011* spot application to the susceptible zone led to a ∼60% increase in the level of IAA (Fig. 3, C and D). This increase was not observed in the 5 mm segment above. Additionally, the spot-inoculated susceptible zone of roots pretreated with PPBo for 24 h also did not show an increase in IAA levels (Fig. 3D), suggesting that the observed increase in IAA in the susceptible zone is likely due to local YUC activity.

To test whether addition of 10 *μ*M PPBo prior to inoculation affects nodulation, we transferred 3-day-old Medicago seedlings to plates containing either 10 *μ*M PPBo or mock. After 24 h, the root susceptible zones of these seedlings were spot-inoculated with *Sm2011*, and at 7 dpi, nodule numbers were scored. This analysis showed that in mock-treated plants, spot inoculation resulted in nodule formation at the site of rhizobia application approximately 96% of the time (46 out of 48). In contrast, when roots were pretreated with PPBo before spot inoculation, the nodule formation rate dropped to below 5% (3 out of 62; Fig. 3E). Although PPBo application led to a slight reduction of the primary root growth, it had no effect on the number of lateral roots formed on these treated plants, while almost completely blocking nodulation (Supplementary Fig. S6, A to C).

Apart from blocking rhizobia-induced IAA accumulation in the susceptible zone, 10 *μ*M PPBo also reduced auxin levels in the Medicago root tip (Fig. 3B). Therefore, we questioned, is IAA biosynthesis at the root tip contributing to the auxin levels required for nodulation in the susceptible zone? For this reason, we removed the Medicago root tip (3 to 5 mm) a few minutes prior to *Sm2011* spot application. Although a relatively harsh treatment, surprisingly it did not affect nodulation compared to the intact roots (Supplementary Fig. S7).

To further investigate the relation between local auxin biosynthesis and nodulation, we created composite Medicago plants bearing transgenic roots in which *MtYUC*s expression was targeted by *RNAi*. It has been shown that in Arabidopsis, the 11 YUC genes have strong functional redundancies, and only higher-order *yucca* mutants display developmental defects (Cheng et al. 2006, 2007). For this reason, we targeted all 3 early induced *MtYUCs* (i.e. *MtYUC1*, *2*, and *8*) simultaneously (*YUCi*). We first used the *35S* promoter to drive this construct. The expression of *MtYUC1*, *MtYUC2*, and *MtYUC8* was reduced by 50 to 60% in *35S::YUCi* transgenic roots compared to an *empty vector* (*EV*) control (Supplementary Fig. S8). At 3 weeks postinoculation (wpi), nodulation in these transgenic roots was reduced by roughly 80% when compared to the *EV* control roots (Fig. 3F). Next, we used the *EARLY NODULIN40* (*ENOD40*) promoter to drive the *YUCi* construct. The *ENOD40* promoter is specifically active in the pericycle prior to nodule initiation (Compaan et al. 2001) and is further activated in the pericycle within 3 h of Nod factor application (Xiao et al. 2014; van Zeijl et al. 2015). At 3 wpi, nodulation on *ENOD40:YUCi* transgenic roots was reduced by roughly 65% compared to the *EV* control roots (Fig. 3G).

**Figure 4.**
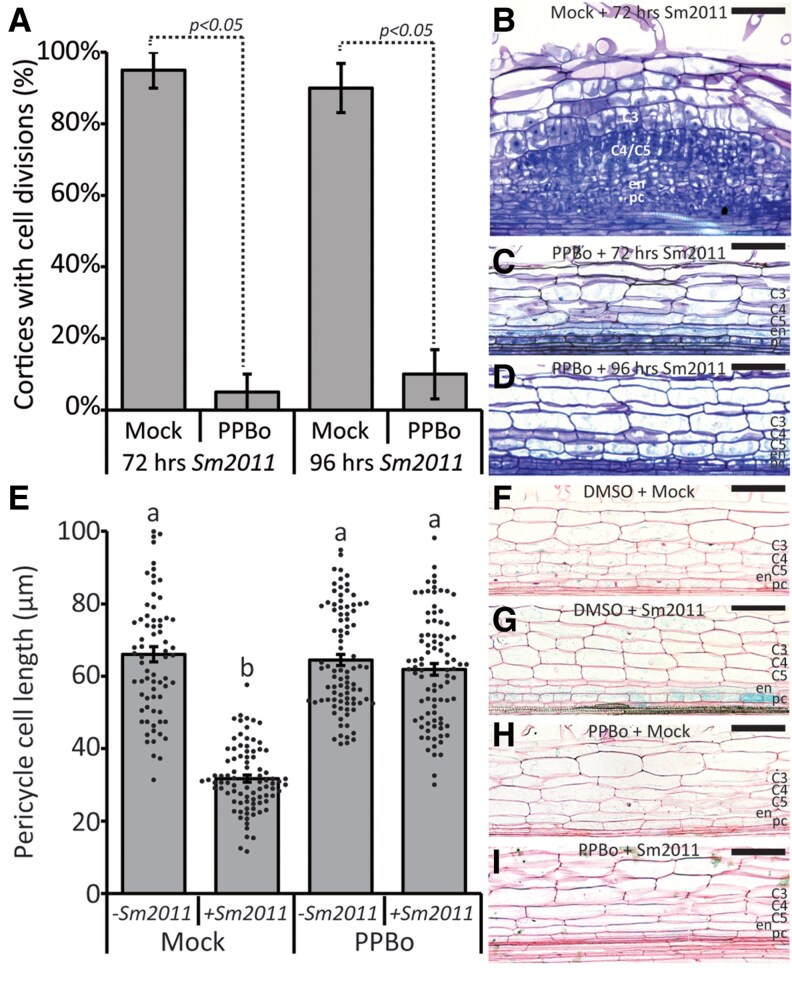
The effect of pharmacological inhibition of *Mt*YUCCA on nodule initiation. **A)** Percentage of *Sm2011*-spotted roots showing mitotic reactivation of cortical cells in the 0 *µ*M (mock) or 10 *µ*M 4-PPBo at 72 or 96 h post spot application. Bars represent averages, ± standard error, *P* < 0.05 according to Student's *t*-test, *n* = 20. **B** to **D)** Representative images of the Medicago roots after spot inoculation: **B)** a mock-treated +*Sm2011* for 72 h, **C)** a 10 *µ*M PPBo-treated +*Sm2011* for 72 h, and **D)** a 10 *µ*M PPBo-treated *Sm2011* for 96 h. **E)** Average length of pericycle cells in the root susceptible zone at the application site without (*−Sm2011*) or with (*+Sm2011*) *Sm2011* spot application in the absence (mock) or presence (PPBo) of 10 *µ*M PPBo. Bars represent averages, ±standard error, dots show the length of individual pericycle cells, and different letters indicate significant differences with *P* < 0.05 according to ANOVA and Tukey post hoc test (*n* > 100). **F** to **I)** Representative images of the Medicago root susceptible zone at 24 hpi; the blue staining marks the nodule primordium initiation in *pENOD11_pro_::GUS* stable transgenic A17 line. **F)** A 24 h mock-treated −*Sm2011*, **G)** a 24 h mock-treated +*Sm2011*, **H)** a 24 h 10 *µ*M PPBo-treated −*Sm2011*, and **I)** a 24 h 10 *µ*M PPBo-treated +*Sm2011*. C3-C5, cortical cell layers; en, endodermis; pc, pericycle; scale bars 100 *μ*m.

So far, we have shown that *MtYUC1*, *2*, and *8* are all expressed in the pericycle of the root susceptible zone in response to rhizobia application. Additionally, we demonstrated that application of PPBo blocks the local auxin biosynthesis. As the first step in this process is the induction of pericycle divisions followed by cortical cell divisions, we questioned whether local auxin biosynthesis is critical in either of these cell division processes. Although *MtYUC1*, *2*, and *8* are not expressed in the cortex, we first wondered if interfering with their function in auxin biosynthesis had any effect on the mitotic reactivation of these cortical cells. For this, we pretreated Medicago roots for 24 h with either 0 (mock) or 10 *µ*M PPBo (PPBo) prior to spot inoculation with *Sm2011*. Root segments were collected at 72 and 96 hpi, embedded in plastic and sectioned. This analysis showed that spot application triggered cortical cell divisions in nearly all roots with the mock pretreatment (19 out of 20 [95%] at 72 h and 18 out of 20 [90%] at 96 h). However, in plants pretreated with 10 *µ*M PPBo, cortical cell divisions were observed in only a few cases (1 out of 20 [5%] at 72 h and 2 out of 20 [10%] at 96 h; Fig. 4, A to D). Next, if the root cortex is not mitotically reactivated when pretreated with PPBo, what about the pericycle? As the effect of rhizobia spot application on the pericycle cells already occurs roughly at 24 hpi, we repeated the spot inoculation experiment as described above and collected inoculated root segments at 24 hpi. To facilitate the identification of early initiation events, we used a stable A17 *ENOD11::GUS* line (Journet et al. 2001). GUS signal was readily found in the susceptible zone of mock pretreated plants spotted with *Sm2011*, but not in any of the other treatments (Fig. 4, E to I). To visualize the effect of PPBo application on the mitotic reactivation of the pericycle, we measured the length of the pericycle cells in the root susceptible zone at the application site. This revealed that the pericycle cells pretreated with mock and spotted with *Sm2011* were roughly 50% shorter than those not inoculated (Fig. 4, E to G). This suggest that these cells had undergone a round of cell divisions prior to harvesting. This was not the case in plants pretreated with 10 *µ*M PPBo. In this treatment, *Sm2011* application did not affect pericycle cell length, suggesting that these cells were not dividing (Fig. 4, E to I).

### Spatiotemporal expression of auxin transporters during nodule primordium development

The observation that the auxin biosynthesis genes *MtYUC1*, *2*, *8*, and *9* are not expressed in cortical cells during primordium formation raises the question, “how do these cells acquire the auxin as inferred from *DR5::GUS*?”. As no other *MtYUCs* were detected during nodule primordium development, an obvious answer to this question is polar auxin transport. Polar auxin transport requires the presence of auxin export carrier *Mt*PIN proteins, positioned on the plasma membrane outwards to direct auxin flow from the pericycle, via the endodermis toward the cortex. To investigate this, we first determined the spatial expression pattern of the early induced *MtPIN2*, *MtPIN4*, and *MtPIN10* (Plet et al. 2011; van Zeijl et al. 2015; Kohlen et al. 2018; Schiessl et al. 2019).

**Figure 5.**
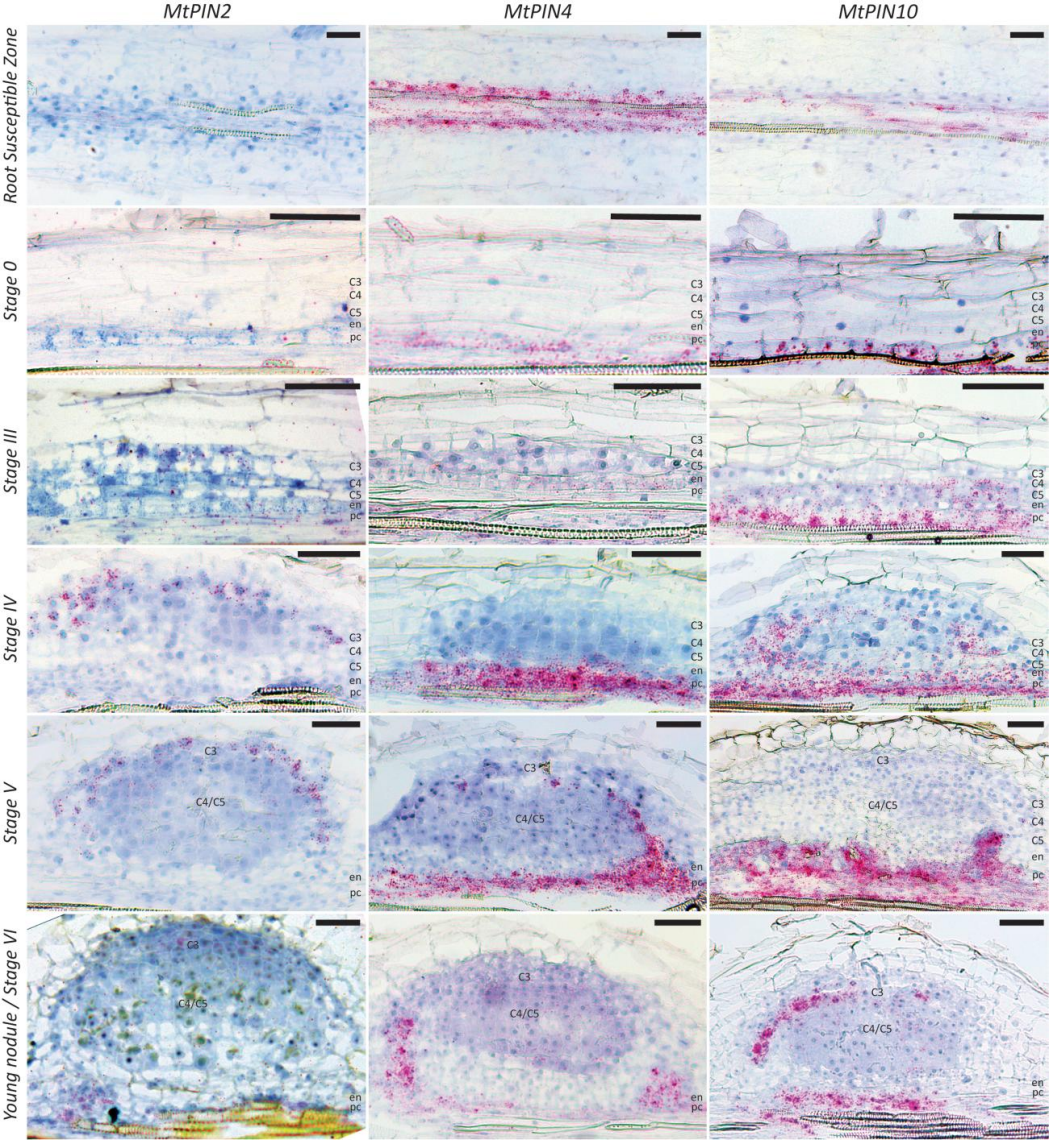
The spatiotemporal expression dynamics of Medicago *PIN-FORMED* (*MtPINs*) in the root susceptible zone and during nodule primordium formation. Representative images of RNA in situ hybridizations with *MtPIN2*, *MtPIN4*, or *MtPIN10* probe sets on longitudinal sections of root segments and nodule primordia at different stages of development. Magenta dots are hybridization signals. In addition, for the *MtPIN2* transcript detection at Stages 0 and III, 2-plex RNA in situ was used that included a Medicago *NUCLEAR FACTOR Y, SUBUNIT A1* (*MtNF-YA1*) probe set Type 6 as a marker for nodule primordium initiation (light blue hybridization signals). C3-C5, cortical cell layers; en, endodermis; pc, pericycle; scale bars 75 *μ*m.

In the root susceptible zone, hybridization signals were only observed for *MtPIN4* and *MtPIN10*, in the stele (Fig. 5), which agrees with the reported expression levels of these genes (van Zeijl et al. 2015). *MtPIN2* transcripts were not detected in the root susceptible zone. To exclude inefficient *MtPIN2* probe set affinity, we used for RNA in situ hybridization longitudinal sections of root tips as a control. In the root tip, *MtPIN2*, *4*, and *10* displayed specific spatial expression patterns (Supplementary Fig. S9), to some extent matching the expression domains of their Arabidopsis putative orthologs. This demonstrated that *MtPIN2* probe set is capable of detecting *MtPIN2* transcripts and that this gene is likely not expressed in the root susceptible zone.

Next, we studied the spatiotemporal expression pattern of *MtPIN2*, *MtPIN4*, and *MtPIN10* at different developmental stages of nodule primordium development by RNA in situ hybridization (Fig. 5). At Stages 0 to I, *MtPIN2* was not visible in the pericycle. *MtPIN4* was detected in the vasculature and pericycle, and this expression pattern was not different from that in the susceptible zone of uninoculated plants (Fig. 5). *MtPIN10* transcripts were observed in root vasculature and with highest intensity in the pericycle, suggesting that *MtPIN10* expression was induced in the pericycle at the start of nodule primordium initiation.

At Stages III to IV, *MtPIN2* transcripts were seen in both dividing and nondividing cortical cells, as well as in the developing nodule vasculature. At Stages V to VI, *MtPIN2* is expressed mainly in the nodule vasculature and the future nodule meristem. Different from *MtPIN2*, *MtPIN4* remained restricted to the pericycle at Stages I to III and to the forming vasculature at Stages IV to VI. *MtPIN10* transcripts were observed also in cortex-derived cells at Stage III and at Stage IV at the periphery of the nodule primordium. At later Stages V and VI, *MtPIN4* and *MtPIN10* hybridization signals were visualized mainly in the developing vasculature. Like *MtPIN2*, *MtPIN10* transcripts were also detectable in the future nodule meristem at Stage VI.

*MtPIN6* is orthologous to the Arabidopsis *PIN6* and has a unique position within the family of PIN auxin transporters as it displays a dual localization, at the plasma membrane and endoplasmic reticulum (Simon et al. 2016). As a result, *At*PIN6 is believed to mediate both, polar auxin transport and intracellular auxin homeostasis. Additionally, *At*PIN6 has been demonstrated to be involved in organogenesis (Cazzonelli et al. 2013; Simon et al. 2016). By using RNA in situ hybridization, we could not detect *MtPIN6* in the root tip, root susceptible zone, or at early nodule primordium Stages 0 to I. However, *MtPIN6* transcripts were visualized in root transition zone and at later time points of nodule development, when nodule vasculature was initiated (Supplementary Fig. S10).

Apart from auxin efflux, auxin influx is a contributing factor to establish directional auxin transport (Bennett et al. 1996). The auxin import carrier *MtLAX2* is highly induced during the early stages of nodule development (de Billy et al. 2001; Roy et al. 2017; Schiessl et al. 2019). We performed in situ RNA hybridization on the Medicago root, including the root tip and susceptible zone. Our results confirmed previously published expression pattern of *MtLAX2* that was detected in the lateral root cap, in some of columella cells and developing vasculature (de Billy et al. 2001) (Supplementary Fig. S11). In addition, strong *MtLAX2* hybridization signals were detected in the vasculature of the susceptible zone. To resolve *MtLAX2* expression dynamics during nodule primordium formation, we performed RNA in situ hybridization on longitudinal sections of spot-inoculated root segments. This revealed that, at Stage 0, *MtLAX2* transcripts were located in the root vasculature and pericycle (Supplementary Fig. S11). At Stages I, II, and III, *MtLAX2* expression was detected mainly in the pericycle and forming nodule vasculature. At later stages, expression was observed in the future nodule meristem, albeit at very low levels (Supplementary Fig. S11).

Taken together, our spatiotemporal studies on auxin transporter gene expression revealed that *MtLAX2*, *MtPIN4*, and *MtPIN10* could be involved in local auxin accumulation at Stages 0 and I in the pericycle, while *MtPIN2*, *MtPIN6*, and *MtPIN10* could participate in creating the auxin pattern in cortical cells.

### MtPIN10 dynamics during nodule formation

If auxin biosynthesis at Stages I and II of nodule primordium initiation does not occur locally in the cortex and both *MtPIN4* and *MtPIN10* are expressed in the pericycle, it implies that auxin might be produced in the pericycle and actively transported toward the cortex. To determine if cellular localization of the expressed *Mt*PIN proteins could facilitate such transport, we created a *MtPIN10::MtPIN10-eGFP* (*Mt*PIN10-GFP) protein fusion. We attempted to create a similar construct for *Mt*PIN4, but unfortunately without success.

First, we tested whether this construct would mimic the expected expression domains. Compound plants baring *Mt*PIN10-GFP transgenic roots showed a similar expression pattern in the root tip (Supplementary Fig. S12) as observed by RNA in situ hybridization for *MtPIN10* (Supplementary Fig. S9). However, no GFP signal was detected in the susceptible zone for *Mt*PIN10 where this gene is expressed (Supplementary Fig. S12).

**Figure 6.**
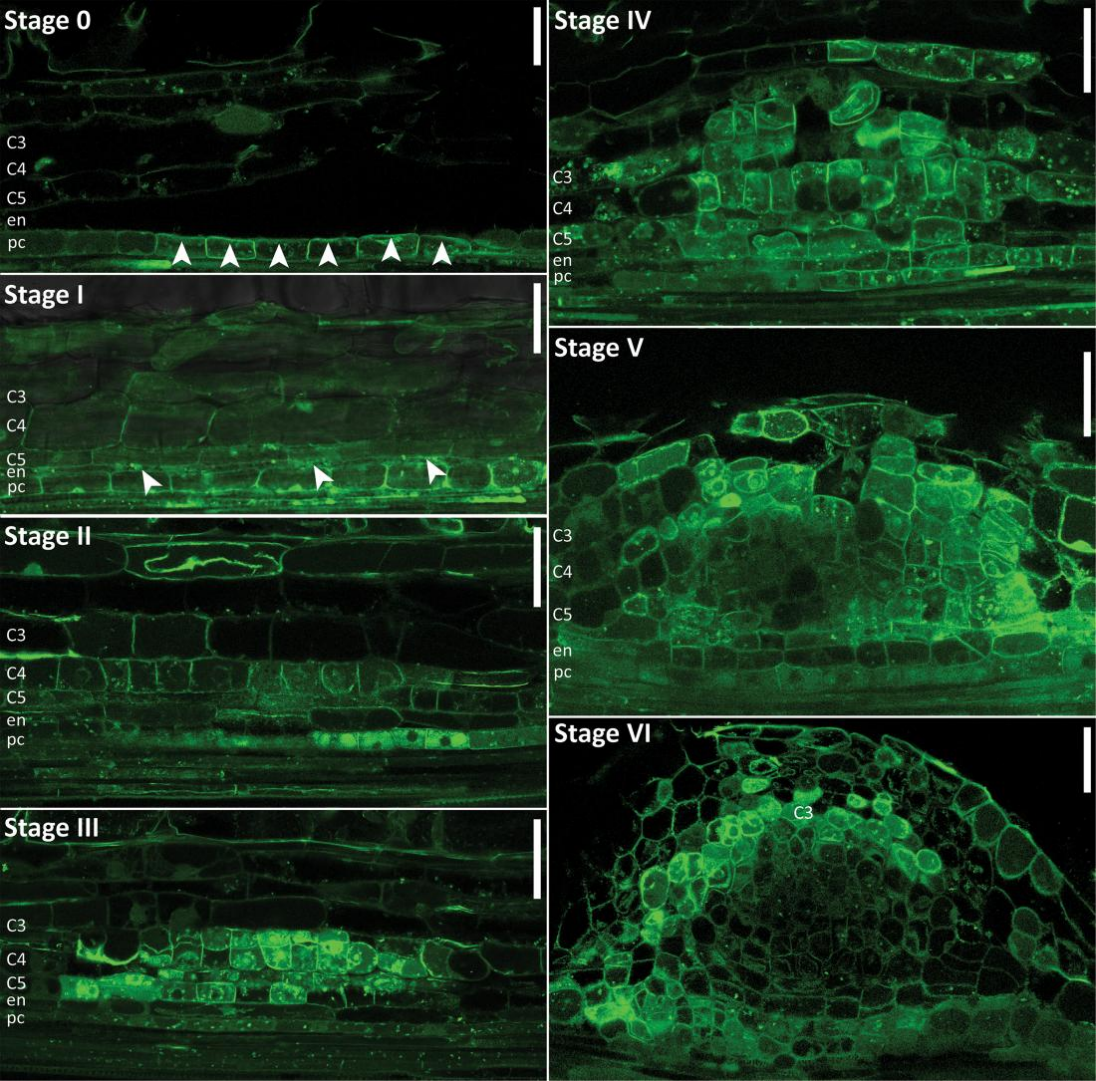
Medicago PIN-FORMED10 (*Mt*PIN10) localization during Medicago nodule primordium formation. Stage 0, *Mt*PIN10-GFP is detected in pericycle cells, partially orientated toward the cortex (arrowheads). Stage I, *Mt*PIN10-GFP is extended to the endodermis (arrowheads). Stage II, *Mt*PIN10-GFP levels are increased in cortical cells with no apparent polarity. Stage III, *Mt*PIN10-GFP is present in pericycle only at low levels. High *Mt*PIN10-GFP levels are detected in dividing cortical cells with no clear polarity. MtPIN10-GFP is also detected in the root vasculature positioned on the plasma membranes toward the root tip. Stage IV, *Mt*PIN10-GFP is positioned toward the center of the primordia in the cells located at the primordium periphery and no clear polarity is visible in the cells located at the center of the primordium. Stage V/VI, *Mt*PIN10-GFP is hardly detectable in the central part of the primordia, at the periphery, in developing nodule vasculature cells, it is positioned toward the (future) nodule meristem. C3-C5, cortical cell layers; en, endodermis; pc, pericycle; scale bars, 75 mm.

Next, we spot-inoculated roots transiently expressing *Mt*PIN10-GFP with *Sm2011*. Root segments were harvested at different time points after inoculation, and longitudinal handmade sections through the vasculature were made and immediately observed under confocal microscope. To determine subcellular *Mt*PIN10 localization, we analyzed ∼5 to 7 primordia for each stage and representative images are shown (Fig. 6).

At Stage 0, MtPIN10 was clearly visible in the pericycle at the site of nodule primordium induction, and, although not exclusively, in part localized toward the endodermis and root cortex (arrowheads). At Stage I, MtPIN10 is extended to the endodermis (arrowheads). At Stage II, MtPIN10 was visible in the endodermis and inner cortex. At Stage III, MtPIN10 was localized in the dividing cortical cells, and later, at Stage IV, the primordium periphery positioned toward the center of the activated cortical cells. At Stages V and VI, MtPIN10 localized in the periphery of the nodule primordia, likely the nodule vasculature. Here, subcellular localization of MtPIN10 was orientated toward the nodule meristem. At these stages, MtPIN10 is hardly detectable in the central part of the primordia.

### Functional analysis of MtPINs during nodulation

A reported Medicago *pin2* mutant did not show a nodulation-related phenotype (Ng et al. 2020). These observations imply that there is a certain functional redundancy of *MtPINs*. Such redundancy is known in Arabidopsis roots where PIN proteins exhibit synergistic interactions when the loss of a specific PIN protein is compensated by auxin-dependent ectopic expression of its homologs (Vieten et al. 2005). Additionally, 2 mutant lines have been isolated and described for Medicago *PIN10*, loss-of-function *pin10/smooth leaf margin1 (slm1)* (Peng and Chen 2011; Zhou et al. 2011a, 2011b). These mutant lines display severe pleiotropic phenotypes in different organs including leaf and flower development. In addition, both lines are sterile. For this reason, these mutants must be maintained as heterozygous, and for neither mutant, a nodule phenotype has been described.

**Figure 7.**
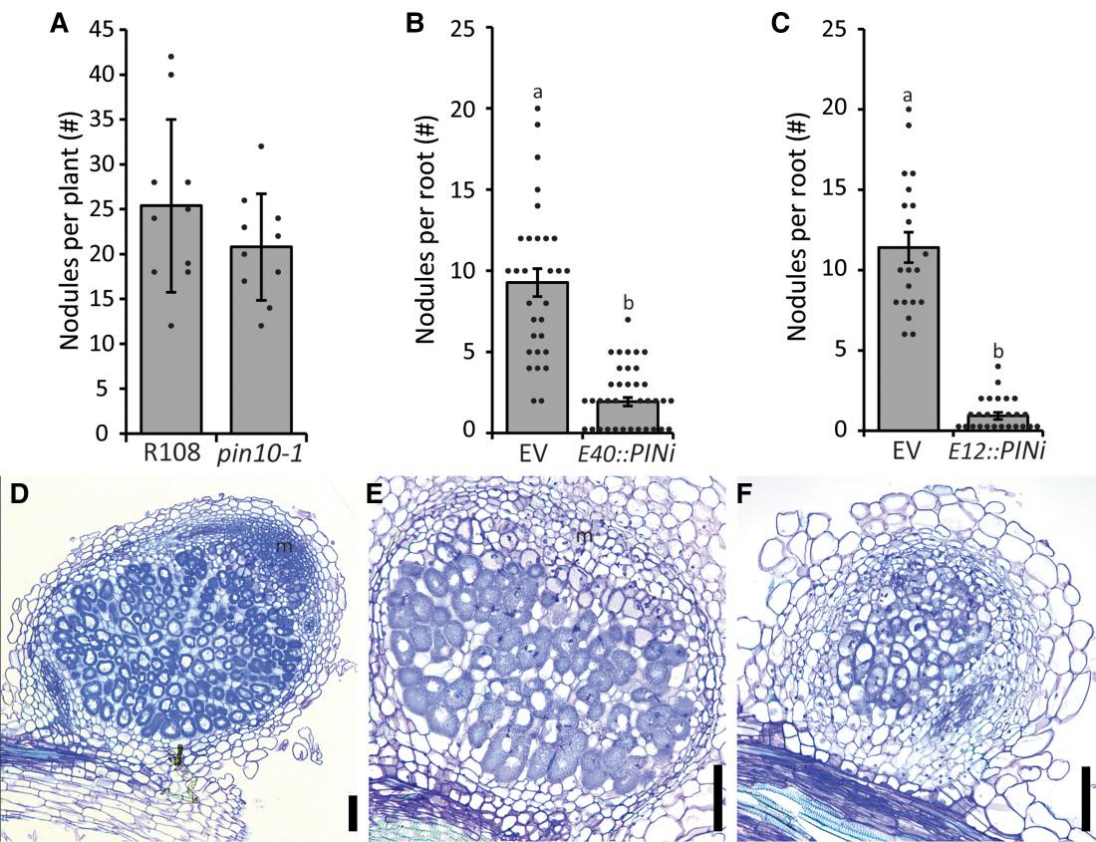
The role of auxin transport during nodulation in Medicago. **A)** The number of nodules formed on roots of Medicago *pin-formed10* (*Mtpin10-1*) compared to wild type (R108). Bars represent averages, ±standard error, dots show nodules per individual root, no significant differences with *P* > 0.05 according to Student's *t*-test (*n* > 13). **B)** The number of nodules formed on *ENOD40_pro_*::*PIN2/4/10i (E40::PINi)* transgenic roots compared to *EV* control roots, bars represent averages, ±standard error, dots show individual data points per transgenic root, and different letters indicate significant differences with *P* < 0.05 according to ANOVA and Tukey post hoc test (*n* > 30). **C)** The number of nodules formed on *ENOD12_pro_*::*PIN2/4/10i (E12::PINi)* transgenic roots compared to *EV* control roots, bars represent averages, ±standard error, dots show individual data points per transgenic root, and different letters indicate significant differences with *P* < 0.05 according to ANOVA and Tukey post hoc test (*n* > 20). **D** to **F)** Representative images of **D)** an *EV* control nodule containing a fully developed nodule meristem, **E)** an *E12:PINi* nodule with a short and underdeveloped meristem, and **F)** an *E12:PINi* nodule without a meristem. **D** to **F)** m, meristem; scale bars, 100 *µ*m.

We inoculated Medicago *pin10-1* mutant plants with rhizobia and analyzed nodules at 3 wpi. The *pin10-1* mutation had no significant effect on the number of nodules formed per plant compared to wild-type R108 (Fig. 7A). This observation suggests that *MtPIN10* either plays no role during nodulation, or its involvement is masked by a functional redundancy. In fact, *MtPIN4* has a similar expression pattern as *MtPIN10* (Fig. 5) and therefore might function redundantly to *MtPIN10*. To test this, we generated *RNAi* construct to knock down *MtPIN2*, *MtPIN4*, and *MtPIN10* simultaneously.

We first used the *35S* promoter to drive the *PINi* construct, reducing expression of all 3 targeted *PINs* by 70% to 90% in transgenic roots (Supplementary Fig. S13A). This led to a severe root phenotype where roughly 70% of all roots formed were less than 3 cm long (Supplementary Table S1). Closer inspection revealed that several of these roots failed to maintain a functional apical root meristem (Supplementary Fig. S13, B to D). Overall, nodulation on roots transformed with the *35S::PINi* construct was reduced by roughly 75% compared to an *EV* control (Supplementary Fig. S13E). Additionally, roots expressing the *35S::PINi* construct formed several small nodules with a short and underdeveloped meristem (Supplementary Fig. S13F) and nodule like bumps (Supplementary Fig. S13G).

Next, we used the *ENOD40* promoter to silence these *MtPIN* genes at the very beginning of nodule initiation (Compaan et al. 2001). The introduction of this construct *in planta* by hairy root transformation reduced nodule number by ∼70% compared to the *EV* control (Fig. 7B). This demonstrates that expression of *MtPIN4* and *MtPIN10* in the pericycle is required for nodule initiation. However, as the *ENOD40* promoter is also active in noninoculated roots (Crespi et al. 1994) and *MtPIN4*, *MtPIN10*, and *MtPIN2* are expressed in the pericycle of the Medicago root tip independent of rhizobia application (Fig. 5), we cannot exclude that downregulation of the expression of these *MtPINs* at this position in the root has an effect on nodulation. Therefore, we silenced *MtPIN4*, *MtPIN10*, and *MtPIN2* under the symbiosis-specific promoter *ENOD12* which is strongly activated upon rhizobial infection in all nodule primordium cells and remains active in nodule meristem and infection zone of mature nodules (Limpens et al. 2005; Li et al. 2021). Introduction of this construct led to a similar reduction in nodule numbers compared to the *ENOD40* promoter (Fig. 7, B and C). Moreover, the few nodules formed were small compared to wild type, and plastic sections revealed that they rarely (7 out of 22 vs 22 out of 25 for the EV control) formed a meristem (Fig. 7, D to F, and Table 1). Combined, this shows that downregulation of PINs under control of the *ENOD40* and *ENOD12* promoters has a similar effect on nodule numbers, but no obvious effect on root development. This suggests that downregulation of these *PINs* under the *ENOD40* prior to rhizobium application might not have a strong additional effect on nodule numbers and that local auxin transport, similar to local auxin biosynthesis, could be essential for nodulation.

**Table 1.** *ENOD12_pro_::PINi* (*E12:PINi*) nodule phenotype

*RNAi* composite plants	Nodules sectioned	Normal	Small or no meristem
*EV*	25	22	3
*E12:PINi*	22	7	15

### No effect of blocking shoot-derived auxin on nodulation

**Figure 8.**
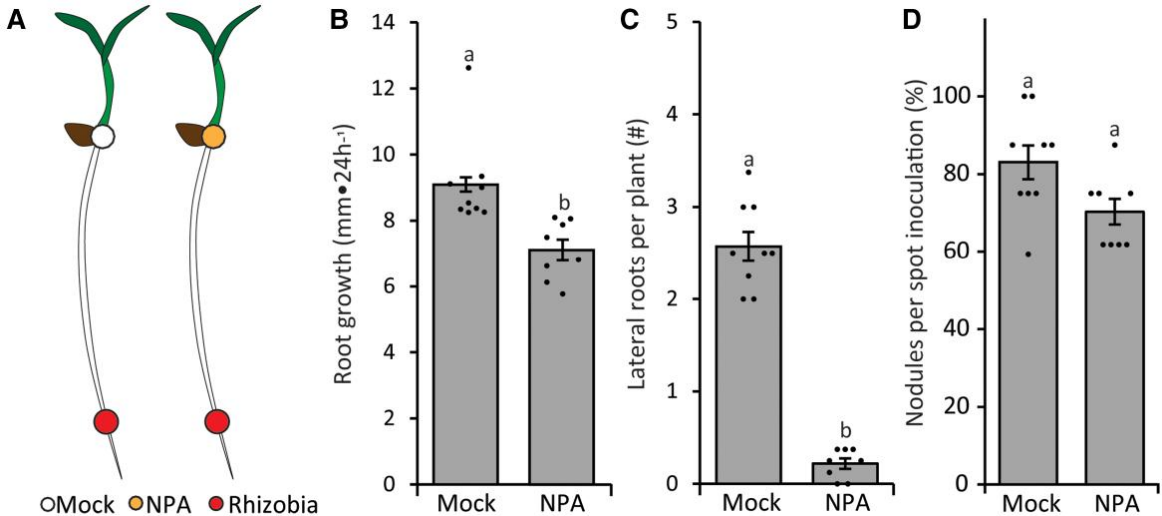
The effect of N-1-NPA application at the hypocotyl–root junction on root development and nodulation. **A)** Schematic representation of the experimental design including location of application of either 0 *µ*M NPA (mock) or 50 *µ*M NPA 24 h prior to *Sm2011* spot application. **B)** Averaged root growth (mm•24h^−1^) at 8 dpi. **C)** The average number of lateral roots per plant. **D)** Percentage of spot-inoculated roots that formed a nodule on the inoculation site. Bars represent averages, ±standard error, dots show average data points per plate, and different letters indicate significant differences with *P* < 0.05 according to ANOVA and Tukey post hoc test (*n* > 20).

It is a well-studied effect that auxin produced in the shoot contributes to the organization of root morphology (McDAVID et al. 1972; Bhalerao et al. 2002; Guo et al. 2005; Overvoorde et al. 2010). Additionally, it was previously demonstrated that polar auxin transport plays a role during nodulation (Mathesius et al. 1998b; Wasson et al. 2006; Deinum et al. 2016). Still, whether auxin produced in the shoot contributes to the formation of root nodules remains an open question. To test if shoot-derived auxin is needed during nodulation, we blocked polar auxin transport at the hypocotyl–root junction using small agar droplets containing either 0 (mock) or 50 *µ*M N-1-naphthylphthalamic acid (NPA; Fig. 8A). To validate that NPA application at the hypocotyl–root junction was affecting the plant roots, we scored additional auxin-related root phenotypes previously linked to shoot-derived auxin such as primary root growth and lateral root initiation (Reed et al. 1998; Bhalerao et al. 2002; Overvoorde et al. 2010; Roychoudhry and Kepinski 2022). This revealed that primary root growth was mildly, but significantly, reduced (∼20%; Fig. 8B) and lateral root development was blocked almost completely (Fig. 8C) when NPA was applied at the hypocotyl–root junction compared to the mock application. Nevertheless, NPA application at the hypocotyl–root junction had no significant effect on nodulation (Fig. 8D).

## Discussion

Here, we presented dynamics of genes and proteins involved in auxin biosynthesis and transport and propose their role in establishing the *DR5* expression patterning during sequential stages of nodule formation. From this, the following picture emerges with respect to how the auxin landscape links to nodulation in Medicago; *DR5* activity is concentrated in pericycle cells preceding their first division, and in a wave-like motion, only a few cells wide, the *DR5* activity moves through the endodermal and cortical cells that give rise to the nodule primordium, vasculature, and ultimately the nodule meristem. Based on this *DR5* patterning, 4 major events during nodule development in which auxin likely plays an important role can be defined as pericycle activation, cortex activation, vascularization, and meristem establishment.

### Local auxin accumulation in the pericycle is a prerequisite for nodule initiation

The models presented by Deinum et al. (2012, 2016) gave priority to a local reduction of auxin efflux (PINs) to explain the auxin maximum preceding nodule formation (Xiao 2015). It has been suggested that this reduction in transport capacity leads to a local increase of auxin levels in the pericycle and inner cortex at the site of nodule primordium initiation (Mathesius et al. 1998a, 1998b; Ng and Mathesius 2018). However, a direct comparison between auxin transport inhibition and rhizobia application is difficult as the auxin response patterns and timing of the initiation between these 2 treatments are different (Ng and Mathesius 2018).

We demonstrate that a local auxin transport network of *MtPIN4/10* and *MtLAX2* are expressed in the stele of the root susceptible zone and that *MtLAX2* is highly expressed in the pericycle at the start of nodule initiation. It is possible that together these auxin transporters create a conduit for an auxin flow from the stele into the pericycle. However, our data show that during the first stages of nodule primordium development (Stage 0 to I), the expression of the auxin biosynthesis genes *MtYUC1*, *2*, and *8* is associated with the mitotic reactivation of the few cells in the pericycle that initiate a nodule primordium. Pericycle-specific *RNAi* against these *MtYUCs (ENOD40::YUCi)* and pharmacological applications with the YUCCA inhibitor PPBo demonstrates that local activity of *MtYUC1*, *2*, *and 8*, and therefore likely local auxin biosynthesis, in the pericycle is crucial for nodule initiation and further progression. It was previously reported that in soybean (*Glycine max*), the expression of *GmYUC2a* is associated with determinate nodule development (Wang et al. 2019). Interestingly, the fact that ectopic expression of *GmYUC2* under control of the *35S* promoter severely reduced nodule numbers, similar to the *RNAi* and pharmacological approach used in our study, suggests that during nodulation, the auxin levels likely need to be precisely balanced to form root nodules.

Our findings that the addition of PPBo, while blocking local auxin biosynthesis and nodule initiation, has no effect on the auxin pool originating from the shoot suggest that acropetal auxin transport might be less important in delivering the auxin required for the establishment of the nodule-initiating auxin maximum. Indeed, when blocking acropetal auxin transport at the hypocotyl–root junction, this did not affect nodulation whereas it reduced root growth and severely hampered lateral root development. This indicates that the NPA treatments were successful and that shoot-derived auxin is less important during nodulation.

It was previously reported that also basipetal auxin transport is not necessary for nodulation (Ng et al. 2020). Similarly, removal of the auxin biosynthesis at the root meristem by removing the Medicago root tip also had no effect on nodulation. In Arabidopsis, it was demonstrated that root wounding induced local auxin biosynthesis near the wound site to regulate root regeneration (Matosevich et al. 2020). We cannot exclude a similar process occurs in Medicago; however, in our experiments, the Medicago root regenerated roughly 50% of the time whereas root nodules were formed at the spot inoculation site always, even when the root tips were removed. Nevertheless, our results with those of Ng et al. (2020) indicate that under normal conditions, auxin produced in the root tip is not required for nodulation.

It has been demonstrated that in Arabidopsis, local auxin biosynthesis and long-distance auxin transport are collaborating in regulation of the auxin homeostasis required for main and lateral root growth (Marchant et al. 2002; Laskowski et al. 2008; Chen et al. 2014; Guo et al. 2014; Tang et al. 2017). Our data suggest that a similar mechanism could underlie Medicago primary and secondary root growth and development, but not nodule initiation. On the other hand, it is likely that local auxin transport attenuation is still needed to sustain an auxin maximum created by local auxin biosynthesis in the pericycle upon nodule initiation.

### Pericycle-derived auxin triggers mitotic reactivation of cortical cells

As the expression of *MtYUC1*, *2*, and *8* during the early stages of nodule initiation is restricted to the pericycle, their spatial expression alone cannot explain the *DR5* pattern beyond Stage I. The only other *MtYUC* reported to be expressed during nodule primordia formation is *MtYUC9*. However, this gene is only expressed at later stages of nodulation (Schiessl et al. 2019). Our in situ hybridization experiments demonstrated that *MtYUC9* expression is only detectable from Stage IV onward and only at the periphery of the nodule primordium. Therefore, it is unlikely that the expression of *MtYUC9* is responsible for the auxin required to initiate the cortical *DR5* activity during the preceding Stages II and III.

Previous studies have shown that in *L. japonicus*, the promoters of *MtYUC1*, *MtYUC2*, and *MtYUC8* homologs (*LjYUCCA1* and *LjYUCCA11*) are activated in cortex-derived nodule primordia cells during nodule emergence (Shrestha et al. 2021). However, this activation was not observed for the promoters of *MtYUC2* or *MtYUC8* in Medicago (Schiessl et al. 2019). Our RNA in situ hybridization results further confirm that these genes, along with *MtYUC1*, are not expressed in the Medicago cortex during nodule primordia initiation. These findings suggest that fundamental differences in the regulation of local auxin biosynthesis may contribute to the distinct nature of the 2 nodule types. However, we cannot rule out the possibility that these differences simply reflect species-specific adaptations in how auxin is able to regulate nodulation.

Nevertheless, *MtYUCs* are not expressed in the root cortex of the susceptible zone with or without rhizobia application, indicating that the inhibitory effect of PPBo on cortical cell divisions can only be indirect. The most likely place where PPBo can exert its effect on *Mt*YUCs is the pericycle. This suggests that auxin locally biosynthesized in the pericycle has to be translocated into the cortex to mitotically reactivate these cells. This could also explain the observed *DR5* activity in the cortex prior to cell divisions in the absence of *MtYUC* expression. Our results further suggest that the activated pericycle cells could serve as an auxin source for this activation.

Translocation toward the cortex is likely mediated by auxin efflux and influx carriers. *MtPIN4*, *MtPIN10*, and *MtLAX2* are expressed in the pericycle during the early stages of nodule initiation, and at least for *Mt*PIN10 protein, we have shown a localization in the pericycle and endodermis, and we hypothesize that this could create an auxin transport channel from the pericycle through the endodermis into the cortex. *MtPIN2* is activated later, in cortical cell layer 5, possibly taking a role in the transport of auxin between different cortical cell layers. *MtPIN6* is activated even later and is likely associated with the formation of the nodule vasculature. Combined, our results with those of Roy et al. (2017) suggest that *Mt*PIN4 and 10, in concert with *Mt*LAX2, are possible candidates to facilitate this putative auxin flux from the pericycle into the cortex. These findings are in line with previous reports on the effect of PIN1 in soybean where a triple CLUSTERED REGULARLY INTERSPACED SHORT PALINDROMIC REPEATS-CRISPR-ASSOCIATED PROTEIN 9 (CRISPR-CAS9) knockout of *GmPIN1a*, *1b*, and *1c* reduced nodule numbers by approximately 30% (Gao et al. 2021). Different from indeterminate nodules, the initiation of determinate nodules on soybean roots follows a different pattern. There, outer cortical cells are mitotically reactivated first, after which cell divisions progress inwards (Turgeon and Bauer 1982). Although the initiation of these nodule types is different, the fact that also in soybean stacked *pin1* mutants show a reduction in nodule numbers suggests a more conserved role for polar auxin transport during nodulation (reviewed by Kohlen et al. 2018; Gao et al. 2021). Nevertheless, the fact that NPA application can trigger the formation of pseudonodules in the absence of rhizobia several weeks post application (Allen et al. 1953; Hirsch et al. 1989; Scheres et al. 1992; Schnabel and Frugoli 2004; Subramanian et al. 2006; Wasson et al. 2006; Zhang et al. 2009; Rightmyer and Long 2011; Shen et al. 2015; Sańko-Sawczenko et al. 2016) while NPA physically blocks all acropetal, basipetal, and radial auxin transport (Ung et al. 2022) leaves open the possibility that other means of auxin translocation, i.e. through plasmodesmata or diffusion, could play a role during nodulation.

Indeed, it was previously demonstrated that the symplastic fluxes facilitated by plasmodesmata are linked to nodule development (Gaudioso-Pedraza et al. 2018). Additionally, plasmodesmata have been implied to be involved in the transport of several plant hormones, including auxin (reviewed by Tee and Faulkner 2024). It is therefore interesting to investigate the role of plasmodesmata in auxin translocation from the pericycle to the cortex. However, Gaudioso-Pedraza et al. (2018) also demonstrated that symplastic connectivity is required for the transcriptional activation of the key nodulation transcription factor *NIN* and some of its downstream targets. This will complicate the distinction between cause and effect, as almost all of the auxin biosynthesis and transport genes reported in our study are (in)directly dependent on NIN (Schiessl et al. 2019).

### Is the nodule meristem an auxin sink or source?

At Stages V and VI, the expression of *MtYUC1*, *2*, and *8* is confined to the base of the nodule and nodule vasculature, while *DR5* activity is highly concentrated in the C3-derived cortical cells where the nodule meristem is about to be formed. As such, also at this stage, *DR5* activity and local auxin biosynthesis do not fully match.

In Arabidopsis, auxin locally produced by *At*YUC is a principal factor for vascular strand formation (Cheng et al. 2006; Cao et al. 2019). The expression of *MtYUC1*, *2*, and *8* in the developing vasculature indicates that in Medicago, *Mt*YUCs might fulfill a similar role in vascular strand formation. However, the strong *MtYUC1*, *2*, and *8* expression at the base of the nodule, in a cell layer likely derived from the pericycle, does not lead to *DR5* activity in these cells. This could mean 2 things. Either, as the synthetic auxin reporter *DR5* is based on a single auxin response factor binding motif (i.e. motif bound by AtARF1) (Ulmasov et al. 1997), the observed *DR5* activity might not reflect the total auxin dynamics. On the other hand, as *DR5* activity requires auxin to be nuclear localized (reviewed by Yu et al. 2022), the absence of a strong *DR5* signal here could therefore imply that most of the auxin produced in the cells at the base of the nodule is immediately transported away. A putative auxin flow toward the forming nodule meristem would explain the *DR5* activity observed in the C3-derived cells. It is possible that auxin, biosynthesized in the base of the nodule, the nodule vascular bundles, or even from the acropetal auxin transport stream, is translocated through the nodule vasculature toward the future nodule meristem.

*MtPIN2*, *4*, and *10* genes are expressed in the nodule vasculature and therefore likely candidates to function in such auxin transport. Medicago mutants that fail to develop proper nodule vascular bundles as *Mtnoot1/2* (Magne et al. 2018; Shen et al. 2020) or *Mtlin4* (Guan et al. 2013), while being impaired in nodule meristem development or maintenance, are supporting the hypothesis that nodule vascular bundles are involved in nodule meristem development or maintenance.

If so, this would suggest that the auxin content of the nodule meristem is regulated through transport of auxin produced elsewhere. We demonstrated that, when the level of *MtPIN2*, *4*, and *10* is reduced during nodule initiation, possibly in the forming nodule vasculature, the formation of the nodule meristem is hampered. Combined, this suggests that auxin transport is needed for the nodule meristem to be formed, and as a consequence, the nodule meristem is likely an auxin sink. This auxin can either be transported directly through the C5-C4 layers, or through the developing nodule vasculature.

Based on the canalization hypothesis, the latter is the most likely option. This hypothesis states that a high source of auxin finds its way to an auxin sink, and in doing so paves the way for the formation of a vascular strand (reviewed by Hajný et al. 2022). During leaf vascular patterning, the tip of the leaf primordia is an auxin source, and as a consequence, vascular strand development progresses from the tip of the leaf toward the base (Scarpella et al. 2006; reviewed by Biedroń and Banasiak 2018). If the nodule meristem is indeed an auxin sink, this canalization hypothesis implies that vascularization would progress from the base of the nodule toward the nodule meristem, which would be in contrast to leaf vascularization. Such a nodule meristem could be considered a nonautonomously acting meristem.

In conclusion, our data suggest that a precise spatiotemporal regulation of auxin outputs is guiding multiple stages of nodule primordium initiation and development. This spatiotemporal regulation is likely the result of a balanced interplay between local auxin biosynthesis and transport. We propose that during the early stages of nodule initiation, auxin is biosynthesized in the pericycle, from where it is translocated into the cortex to mitotically reactivate the cells in the C4 and C5 layers. At later stages, when the auxin flow from the pericycle into cells derived from these C4 and C5 layers diminishes, these cells stop dividing and start to differentiate. At the same time, auxin biosynthesized elsewhere (possibly in the pericycle, developed nodule vasculature and/or acropetal auxin transport stream in the main root) is needed to initiate meristem formation and later ensure its persistence. At this moment, the nodule meristem is a sink, draining the surrounding cells of their auxin. Proximal to the nodule meristem, this auxin reduction triggers cell differentiation leading to the infection zone.

## Materials and methods

### Plant material and bacterial strains

Medicago (*M. truncatula*) R108 seedlings were used to make the stable *DR5::GUS* transgenic line by using *Rhizobium radiobacter* (formerly *Agrobacterium tumefaciens*) (strain AGL1) according to the protocol described by Chabaud et al. (2003). Medicago Jemalong A17 (A17) plants were used to generate *R. rhizogenes* (formerly *Agrobacterium rhizogenes*) (strain MSU440) mediated transgenic roots as previously described by Limpens et al. (2004) for RNAi and protein GFP fusion constructs. For RNAi, 30 to 40 seedlings were transformed with each construct per individual experiment. Each experiment has been repeated at least 3 times. Transgenic roots were separately collected and individually scored for nodule numbers. *Mtpin10-1/slm1* (NF3969) were previously described by Zhou et al. (2011a). Surface sterilization and germination of Medicago seeds were performed as previously described by Limpens et al. (2004). *Sm2011* was used to induce root nodule formation on seedlings growing in perlite or on plates with low nitrate Fä medium (Fähraeus 1957). For spot inoculation, germinated A17 seeds were transferred to square Petri dishes on a filter paper placed on Fä medium with low nitrate (0.25 mm) and 1.5% agar. Plants were subsequently grown vertically for 3 days. Spot inoculation was done on root hairs of susceptible zone under binocular macroscope using a droplet of 0.3 *μ*L *Sm2011* suspension in Fä medium (OD_600_ = 0.02) and marked by puncturing the filter paper alongside the site of inoculation (Schiessl et al. 2019). One micromolar AVG was added to plates growing R108 plants only.

### Constructs

DNA fragments were amplified from Medicago genomic DNA using primer combinations listed in Supplementary Table S2 and Phusion High*-*Fidelity DNA Polymerase (Finnzymes). To create the constructs, pENTR/D-TOPO Cloning Kits (Invitrogen) and Gateway technology (Invitrogen) were used to generate the entry clones for RNAi constructs, promoter-GUS and PIN10-GFP constructs. First, 14 synthetic DR5 DNA fragments (Ulmasov et al. 1997) were introduced in the entry clone. Then, the entry vector was recombined into Gateway-compatible binary vector pKGW-RR that contains the GUS reporter gene and *AtUBQ10::DsRED1* as a selection marker (Limpens et al. 2004), by using Gateway LR Clonase II enzyme mix (Invitrogen). PCR fragments of about 400 to 600 bp of single *MtYUCs* and *MtPINs* genes for RNAi constructs were generated on cDNA made from Medicago nodule or root RNA. These fragments were combined by subsequential PCR steps using primers with a complementary 15 bp overhang to generate 1 amplicon of 2 or 3 *DNA* fragments. The final DNA fragments were cloned into pENTR-D-TOPO and recombined into the Gateway-compatible binary vector pK7GWIWG2(II)-UBQ10::DsRED driven by 2 different nodule-specific promoters, *ENOD12* or *ENOD40*, or *CaMV 35S* (*35S*) promoter (Compaan et al. 2001; Limpens et al. 2004) to create the final RNAi constructs. The control for *RNAi* constructs was *ENOD12::EV* containing no coding DNA sequences. For *Mt*PIN10-GFP fusion constructs, the promoter and first part of the DNA fragment was introduced into Gateway donor vector pENTR2-1, eGFP DNA fragment without start codon were introduced into pENTR1-2, and the rest of the gene including the terminator was introduced into pENTR2-3, using Gateway BP Clonase II enzyme mix. These entry vectors were recombined into Gateway-compatible binary vector pKGW-RR-MGW containing *AtUBQ10::DsRED1* as a selection marker using Gateway LR Clonase II Plus enzyme mix (Invitrogen). Insertion sites for the eGFP reporter in *PIN* genes were selected based on functional AtPINs::GFP protein fusion constructs in Arabidopsis (*A. thaliana*) (Xu and Scheres 2005), which are at position 1237 and 1565 for *MtPIN2* and *MtPIN10*, respectively. All used primers are listed in Supplementary Table S2.

### Tissue embedding, sectioning, and staining

Root segments were fixed in 4% paraformaldehyde (w/v), 5% glutaraldehyde (v/v), 0.05 m sodium phosphate buffer (pH7.2) at 4 °C overnight. The fixed material was dehydrated in an ethanol series and subsequently embedded in Technovit 7100 (Heraeus Kulzer) according to the manufacturer's protocol. Sections (7 *µ*m) were made by using a RJ2035 microtome (Leica Microsystems, Rijswijk, The Netherland), stained 5 min in 0.1% Ruthenium Red (Sigma, Germany). Sections were analyzed by using a DM5500B microscope equipped with a DFC425C camera (Leica Microsystems, Wetzlar, Germany).

### Suppression of YUCCAs by 10 *μ*M PPBo

The germinated Medicago seeds were placed on Fä plates for 4 days. These seedlings were either flushed with 1 mL liquid Fä medium containing 10 *μ*M 4-PPBo or mock for 3 h, or transferred to new plates containing Fä medium with either 10 *μ*M PPBo or mock (Fähraeus 1957; Kakei et al. 2015). The flushed roots were harvested to assess the effect of PPBo on auxin biosynthesis in the Medicago root. Roots of the transferred seedlings were let to recover for 24 h and subsequently spot-inoculated with *Sm2011* (OD_600_ = 0.02). Plants were grown further for 1, 2, 3, 4, or 7 more days depending on the experiment.

### Suppression of polar auxin transport by 50 *μ*M *NPA*

The germinated seeds were placed on Fä plates for 4 days and then treated with NPA. NPA (50 mm) was dissolved in DMSO and diluted to an end concentration of 50 *µ*M in Fä medium with 0.8% Daishin agar. Two hundred microliters agar droplets containing either NPA or mock (0.1% DMSO) were solidified and locally applied on the hypocotyl–root junction. After 24 h, roots were spot-inoculated with *Sm2011* (OD_600_ = 0.02). Plants were grown further for 8 days to form nodules. Successful nodulation, root growth, and the number of lateral roots were assessed.

### Auxin quantification

Auxin (indole-3-acidic acid [IAA]) was quantified through Multi Reaction Monitoring Liquid Chromatography-tandem Mass Spectrometry (MRM-LC-MS/MS). Medicago roots were collected and flash frozen in liquid nitrogen. Collected tissue was ground to a fine powder at −80 °C using 3 mm stainless steel beads at 50 Hz for 2*30 s in a TissueLyser LT (Qiagen, Germantown, USA), and between 10 and 15 mg of ground tissue per sample was used for IAA extraction. Samples were extracted with 1 mL of cold methanol containing [phenyl ^13^C_6_]-IAA (0.1 nmol/mL) as an internal standard in a 2 mL Eppendorf tube and purified as previously described (Ruyter-Spira et al. 2011). Samples were filtered through a 0.45 *μ*m Minisart SRP4 filter (Sartorius, Goettingen, Germany) and measured on the same day. Auxin was analyzed on a Waters Xevo TQs tandem quadruple mass spectrometer as previously described (Ruyter-Spira et al. 2011; Gühl et al. 2021).

### RNA isolation and RT-qPCR

RNA was isolated from transgenic *35S_pro_::YUC1/2/8i* (*35S::YUCi*), *35S_pro_::PIN2/4/10i* (*35S::PINi*), and *EV* root tips (5 to 7 mm) using RNA Easy kit (Qiagen). Five hundred nanograms RNA was used for cDNA synthesis using the iScript cDNA synthesis kit (Bio-Rad). qRT-PCR was performed using SYBR Green Supermix and CFX real-time system (Bio-Rad). Gene expression was normalized using *ACTIN2* as a reference gene. Primers used for qPCR are listed in Supplementary Table S2.

### RNA in situ hybridization

RNA in situ hybridization was conducted using Invitrogen ViewRNA ISH Tissue 1-Plex and 2-Plex Assay kits (Thermo Fisher Scientific) using the manufacture’s protocol optimized for Medicago root and nodules sections (Kulikova et al. 2018). RNA ISH probe sets were designed and synthesized at Thermo Fisher Scientific. Assay IDs of used probe sets are presented in Supplementary Table S3. For each stage of nodule primordium development and with each probe set, at least 3 in situ hybridizations were performed, each hybridization procedure included 2 to 3 different root segments on a slide. As a negative control for each hybridization run, 1 slide was used without any probe set. Medicago *NUCLEAR FACTOR Y, SUBUNIT A1* (*MtNF-YA1*) probe set Type 6 was used as a marker for nodule primordium initiation (blue hybridization signals). The images were taken with an AU5500B microscope equipped with a DFC425c camera (Leica).

### Detection of MtPIN10-GFP

Transgenic roots or root nodules were manually sectioned in a longitudinal direction, mounted on microscope slides and immediately observed under a Leica SP8 confocal microscope. GFP is visualized by excitation at 488 nm and by detection at 505 to 530 nm. In these experiments, the 488 and 552 nm lasers were used at 14% and 3.8% intensities, respectively. Images were obtained using the HC PL APO 40x/1.1 W CORR CS2 (#506425) objective, 488/522 Beamsplitter, and Fluo Turret Scan-BF. HyD 1 gain (%) was set at 175.8, PMT 2 gain (V) at 661.1 with 3.56% offset.

### Statistics

When appropriate, data were subjected to the Student's *t*-test (Microsoft Excel). All other data were subjected to 1-way ANOVA. Individual differences were then identified using a post hoc Tukey test (*P* < 0.05). All analyses were performed using SAS_9.20 (http://www.sas.com/). For RT-qPCR, statistical significance was determined based on Student's *t*-test (*P* < 0.01), as implemented in CFX Manager 3.0 software (Bio-Rad, Hercules, USA).

### Accession numbers

Sequences of major genes used in this study can be found at https://medicago.toulouse.inra.fr/MtrunA17r5.0-ANR/ under the following gene ID numbers: *MtNF-YA1* (MtrunA17_Chr1g0177091), *MtYUC1* (MtrunA17_Chr3g0139441), *MtYUC2* (MtrunA17_Chr6g0485621), *MtYUC8* (MtrunA17_Chr7g0262591), *MtYUC9* (MtrunA17_Chr1g0182991), *MtPIN2* (MtrunA17_Chr4g0071571), *MtPIN4* (MtrunA17_Chr6g0478431), *MtPIN6* (MtrunA17_Chr1g0159341), *MtPIN10* (MtrunA17_Chr7g0255941), and *MtLAX2* (MtrunA17_Chr7g0241841) (for Mt4 gene IDs, see Supplementary Table S3).

## Supplementary Material

kiaf133_Supplementary_Data

## Data Availability

Data available on request.

## Supplementary data

The following materials are available in the online version of this article.

**Supplementary Figure S1.**  *DR5::GUS* expression pattern in Medicago roots of R108 and A17.

**Supplementary Figure S2.**  *DR5::GUS* expression pattern in Medicago R108 nodule.

**Supplementary Figure S3.** Dynamics of *DR5::GUS* expression patterns during nodule development in Medicago A17.

**Supplementary Figure S4.** The spatiotemporal expression patterns of *YUCCAs* in root tip of Medicago A17.

**Supplementary Figure S5.** The spatiotemporal expression patterns of *MtYUC9* during nodule primordium formation in Medicago A17.

**Supplementary Figure S6.** Effect of 4-PPBo application on root development and nodulation.

**Supplementary Figure S7.** Effect of removal of the primary root tip on nodulation.

**Supplementary Figure S8.** Validation of RNA interference on *MtYUC1*, *MtYUC2*, and *MtYUC8* targeted by *YUCi* in composite Medicago A17 plants.

**Supplementary Figure S9.** The spatiotemporal expression patterns of *PINs* in the root tip of Medicago A17.

**Supplementary Figure S10.** The spatiotemporal expression patterns of *MtPIN6* during nodule primordium formation.

**Supplementary Figure S11.** The expression patterns of *MtLAX2* in the root and nodule primordia at different stages of development in Medicago A17.

**Supplementary Figure S12.**  *Mt*PIN10 pattern in the Medicago root.

**Supplementary Figure S13.** Validation of RNA interference on *MtPIN2*, *MtPIN4*, and *MtPIN10* in roots of composite Medicago A17 plants.

**Supplementary Table S1.** Phenotype of 35S::PIN2/4/10 RNAi transgenic roots.

**Supplementary Table S2.** List of primers.

**Supplementary Table S3.** Gene probe sets used for ViewRNA in situ hybridization.

## Dive Curated Terms

The following phenotypic, genotypic, and functional terms are of significance to the work described in this paper:

PIN1 Gramene: AT1G73590
PIN1 Araport: AT1G73590

PIN6 Gramene: AT1G77110
PIN6 Araport: AT1G77110

auxin CHEBI: CHEBI:22676

root AmiGo: PO:0009005
