# Supplementary material for: Nodule organogenesis in Medicago truncatula requires local stage-specific auxin biosynthesis and transport
Source: Plant Physiol. 2025 Apr 4;197(4):kiaf133. doi: 10.1093/plphys/kiaf133 (PMC12002018; doi:10.1093/plphys/kiaf133)
Supplement: kiaf133_Supplementary_Data [file kiaf133_supplementary_data.pdf]

Supplementary Figures

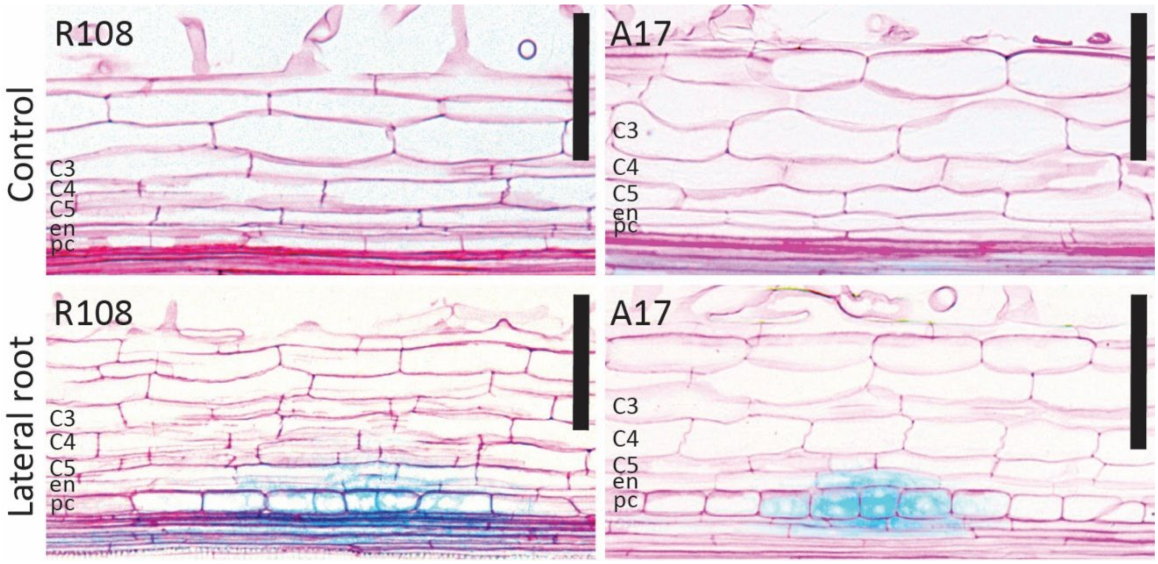

Supplementary Figure S1. *DR5::GUS* expression pattern in Medicago roots of R108 and A17. Representative images of a control root and young lateral root primordia highlighting the narrow initiation site of lateral root development. Longitudinal plastic sections of root segments stained with ruthenium red. C3-C5, cortical cell layers; en, endodermis; pc, pericycle; scale bars 75 µm.

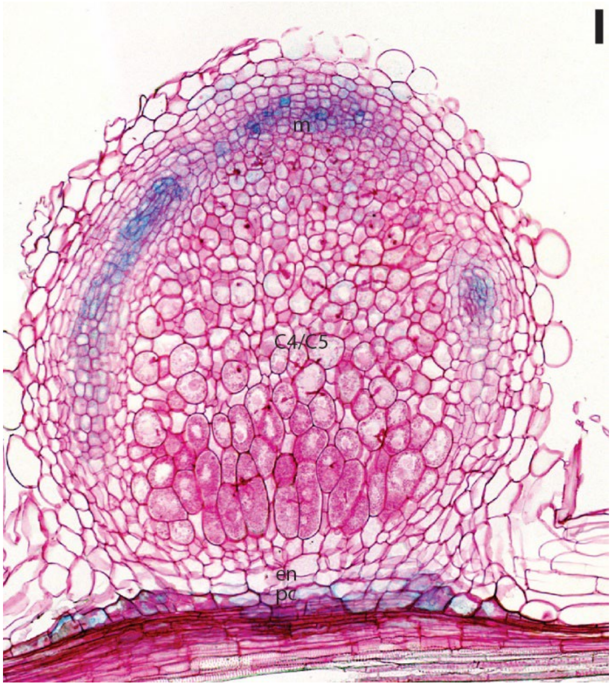

Supplementary Figure S2. *DR5::GUS* expression pattern in Medicago R108 nodule. Longitudinal plastic sections of a mature nodule stained with ruthenium red (C4-C5, cortical cell layers; en, endodermis; pc, pericycle; m, meristem; scale bars 75 µm).

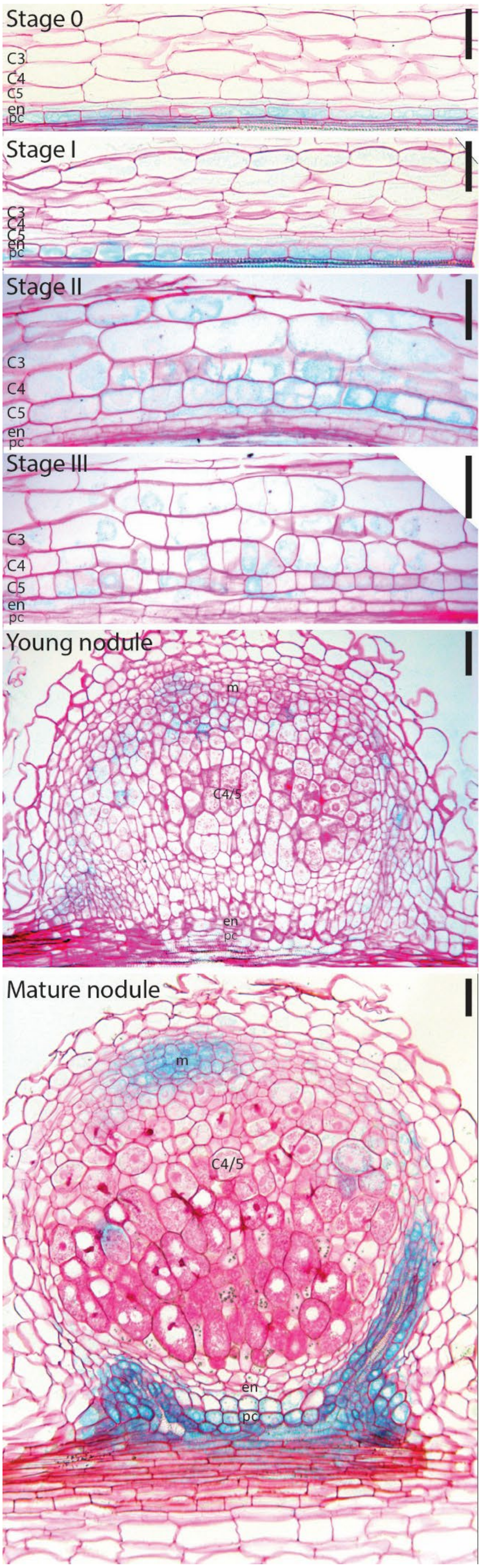

Supplementary Figure S3. Dynamics of *DR5::GUS* expression patterns during nodule development in Medicago A17. The *DR5::GUS* construct was introduced in A17 by transient hairy root transformation. Longitudinal plastic sections of root segments with different stages of nodule development were stained with ruthenium red. C3-C5, cortical cell layers; en, endodermis; pc, pericycle; m, meristem; scale bars 75 µm.

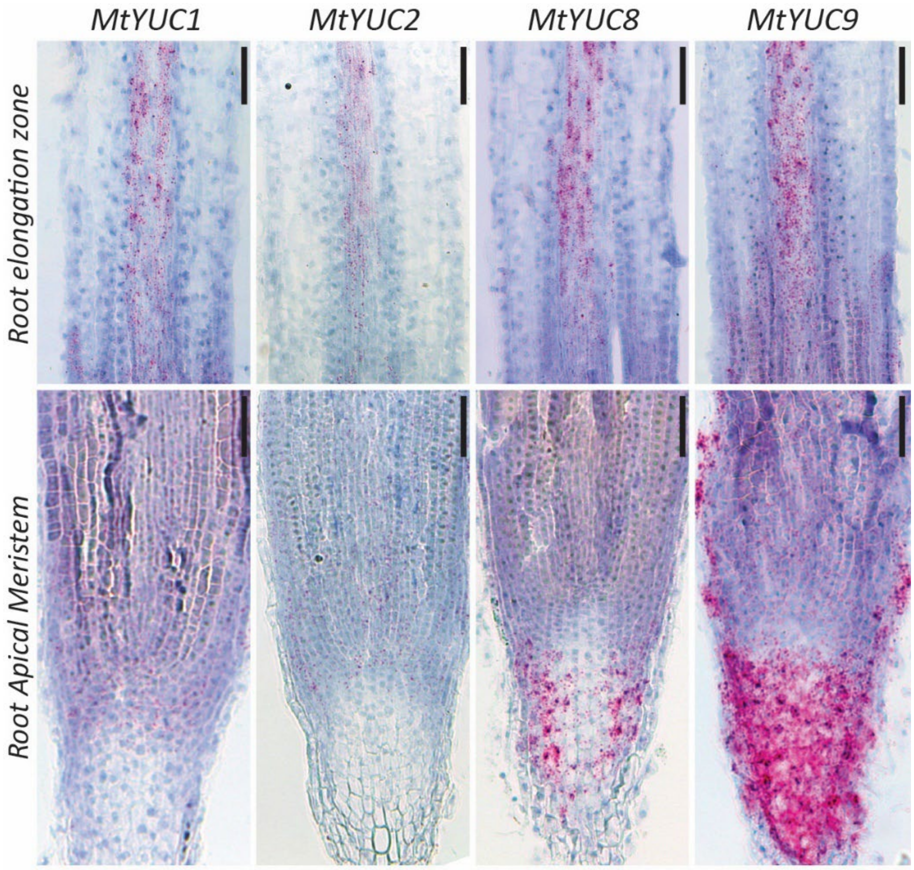

Supplementary Figure S4: The spatiotemporal expression patterns of Medicago *YUCCAs* (*MtYUCs*) in root tip of Medicago A17. Representative images of RNA *in situ* hybridizations with *MtYUC1*, *MtYUC2*, *MtYUC8* or *MtYUC9* probe sets on longitudinal sections of the root tip including elongation zone and root meristem (red dots are hybridization signals; scale bars 75µm).

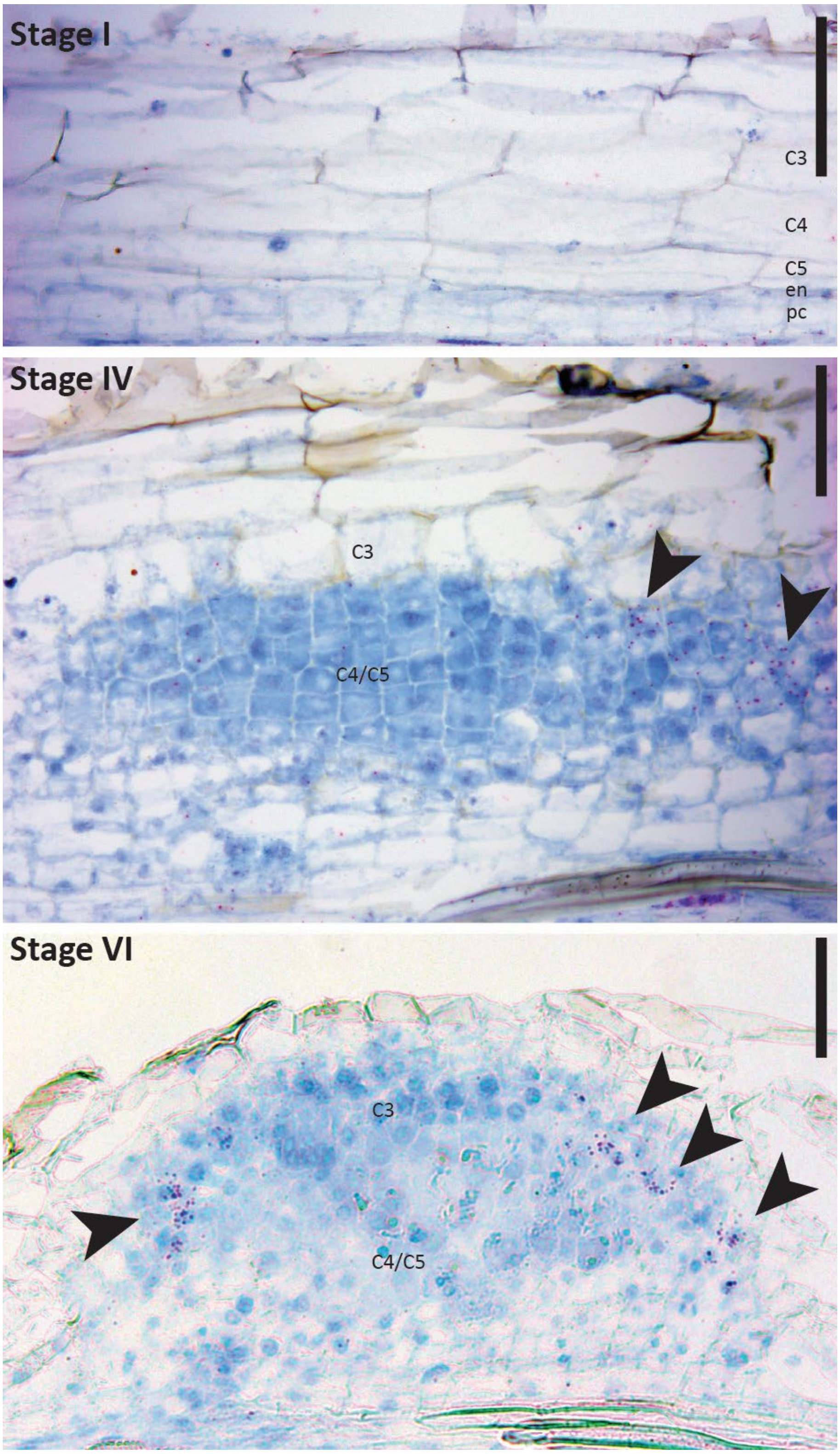

Supplementary Figure S5: The spatiotemporal expression patterns of Medicago *YUCCA9* (*MtYUC9*) during nodule primordium formation in Medicago A17. Representative images of RNA *in situ* hybridization with *MtYUC9* probe set on longitudinal sections of nodule primordia stages I, IV and VI (red dots are hybridization signals; arrows indicate regions with hybridization signals). C3-C5, cortical cell layers; en, endodermis; pc, pericycle; scale bars 75 µm.

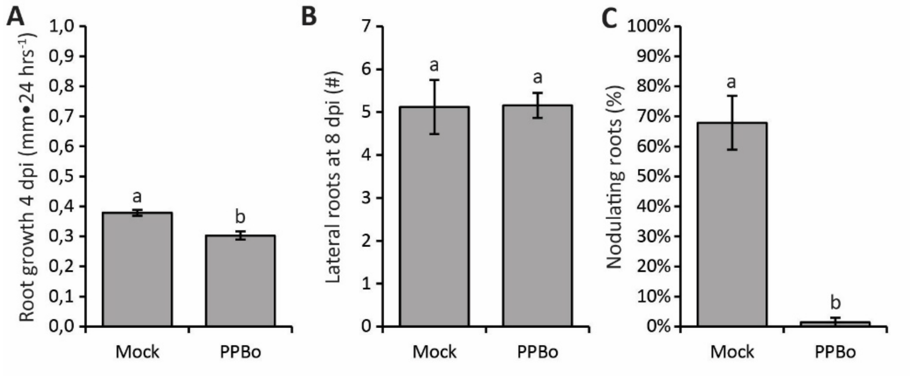

Supplementary Figure S6: Effect of 4-phenoxyphenylboronic acid (PPBo) application on root development and nodulation. **(A)** Averaged root growth (mm•24h^{-1}) at 4 days post inoculation (dpi). **(B)** The average number of later roots per plant. **(C)** Percentage of spot-inoculated roots that formed a nodule on the inoculation site. Bars represent averages, $\pm$ standard error, different letters indicate significant differences with $p < 0.05$ according to ANOVA and Tukey post-hoc test, n>28.

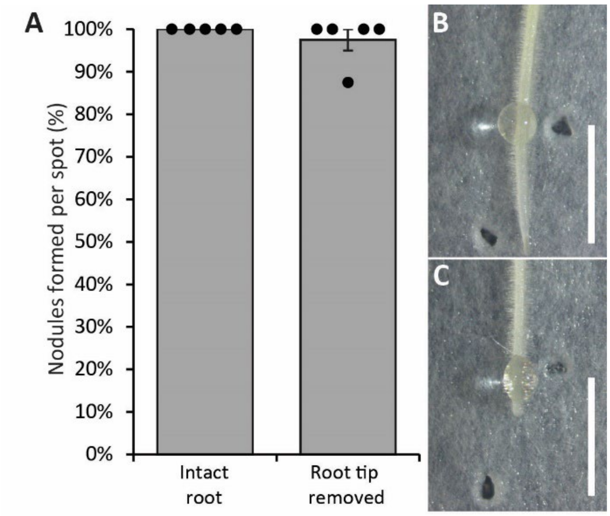

Supplementary Figure S7: Effect of removal of the primary root tip on nodulation. **(A)** Average percentage of nodules formed per plate after rhizobia spot-applications on intact or on Medicago roots with tips removed, bars represent averages, + standard error, dots show percentage of spotted roots to form a nodule per plate, n=5 (5 plates with each ~6-8 Medicago plants). **(B)** Application spot on the intact root, **(C)** Application spot on the root with the root tip removed. Scale bars 5 mm.

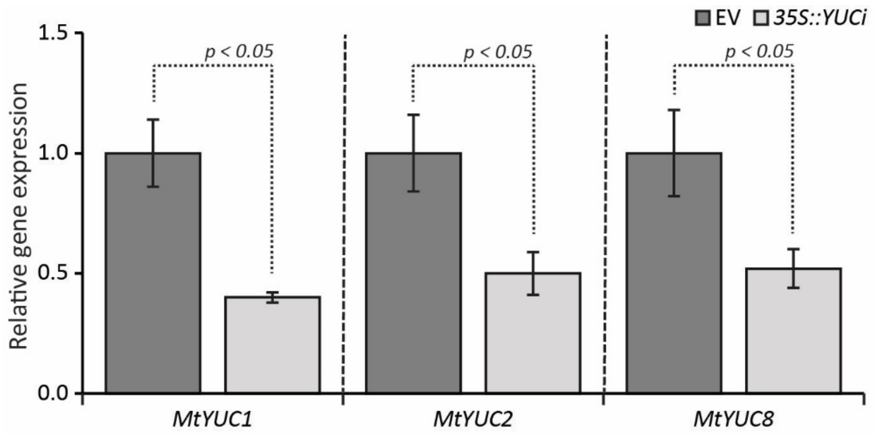

Supplementary Figure S8: Validation of RNA interference on Medicago *YUCCA1* (*MtYUC1*), *MtYUC2* and *MtYUC8* targeted by *YUCi* in composite Medicago A17 plants. Relative expression of *MtYUC1*, *2* and *8* in transgenic $35S_{pro}$*::YUC1/2/8i* (*35S::YUCi*) roots compared to *Empty Vector* (*EV*) control roots, bars represent averages, ± standard error, n=3. Statistical significance was determined based on Student's t-test, $p < 0.05$, as implemented in CFX Manager 3.0 software (Bio-Rad, Hercules, USA).

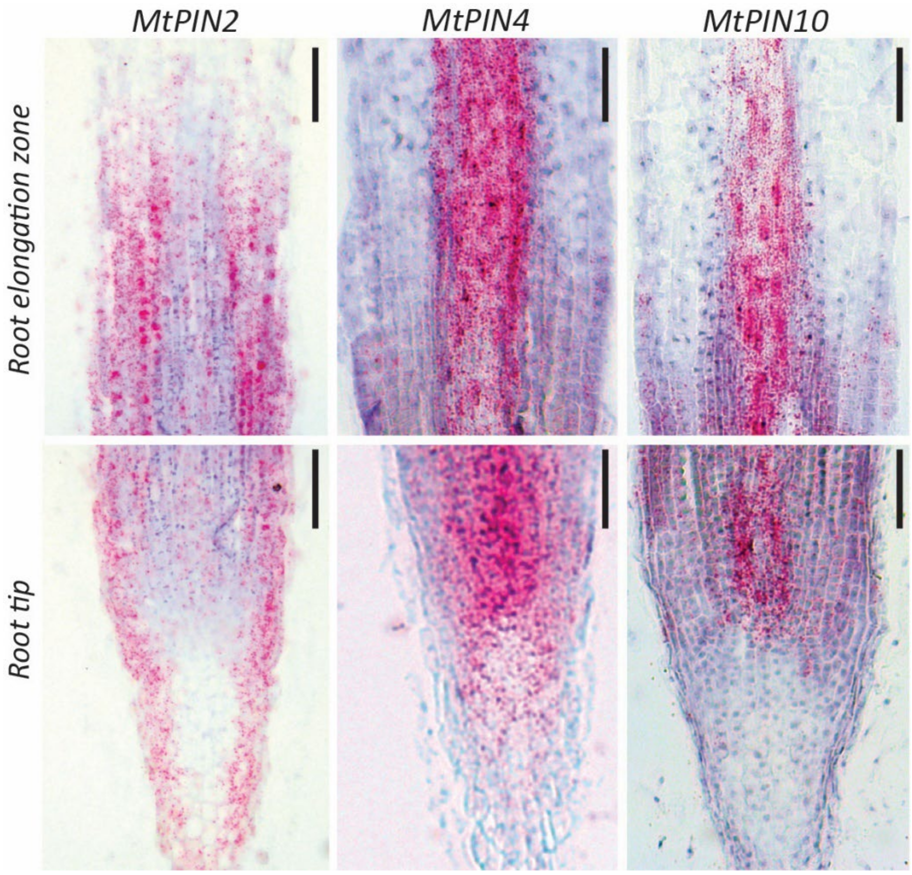

Supplementary Figure S9: The spatiotemporal expression patterns of Medicago *PIN-FORMED* genes (*PINs*) in the root tip of Medicago A17. Representative images of RNA *in situ* hybridizations with *MtPIN2, MtPIN4*, or *MtPIN10* probe set on longitudinal sections of the root tip including elongation zone and root meristem (red dots are hybridization signals; scale bars 75μm).

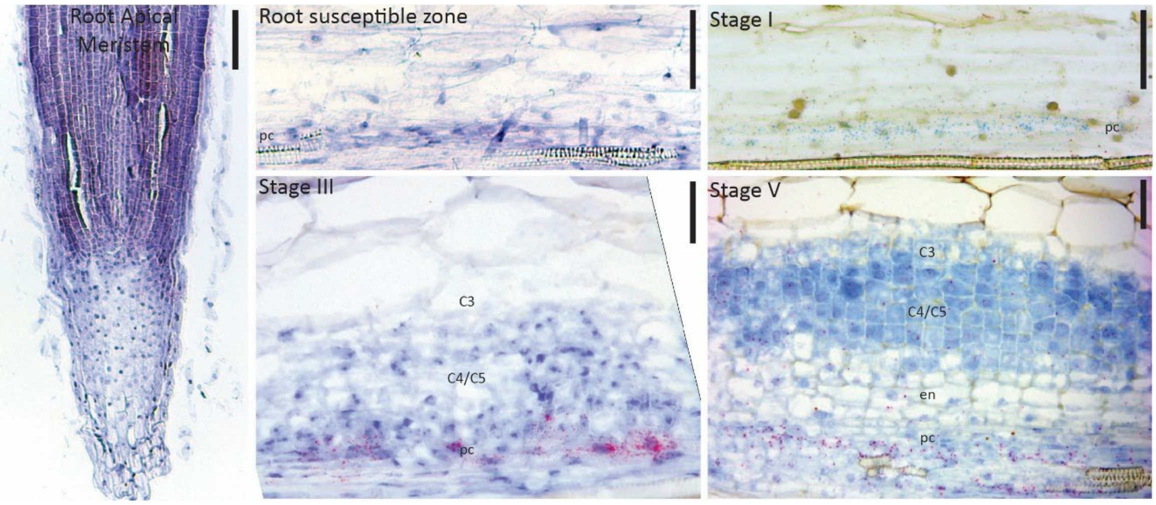

Supplementary Figure S10: The spatiotemporal expression patterns of Medicago *PIN-FORMED6* (*MtPIN6*) during nodule primordium formation. Representative images of RNA *in situ* hybridization with *MtPIN6* probe sets on longitudinal sections of the root tip, the susceptible zone and nodule primordia at stage I, III, and V. For stage I, Medicago *NUCLEAR FACTOR Y, SUBUNIT A1* (*MtNF-YA1*) was used as a marker for nodule primordium initiation. (Red dots are *MtPIN6* hybridization signals, blue dots are *MtNF-YA1* hybridization signals). C3-C5, cortical cell layers; en, endodermis; pc, pericycle; scale bars 75 µm.

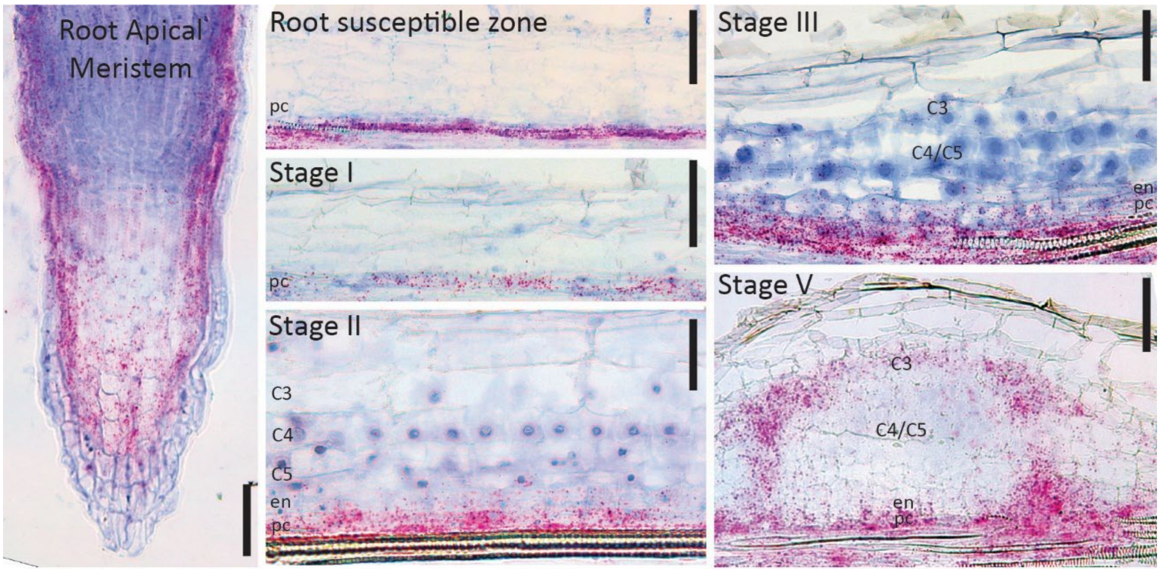

Supplementary Figure S11: The expression patterns of Medicago *LIKE AUXIN RESISTANT2* (*MtLAX2*) in the root and nodule primordia at different stages of development in Medicago A17. Representative images of RNA *in situ* hybridization with *MtLAX2* probe set on longitudinal sections of the root tip, the root susceptible zone, and nodule primordia at stages I, II, III, and V (red dots are hybridization signals). C3-C5, cortical cell layers; en, endodermis; pc, pericycle; scale bars 75 µm.

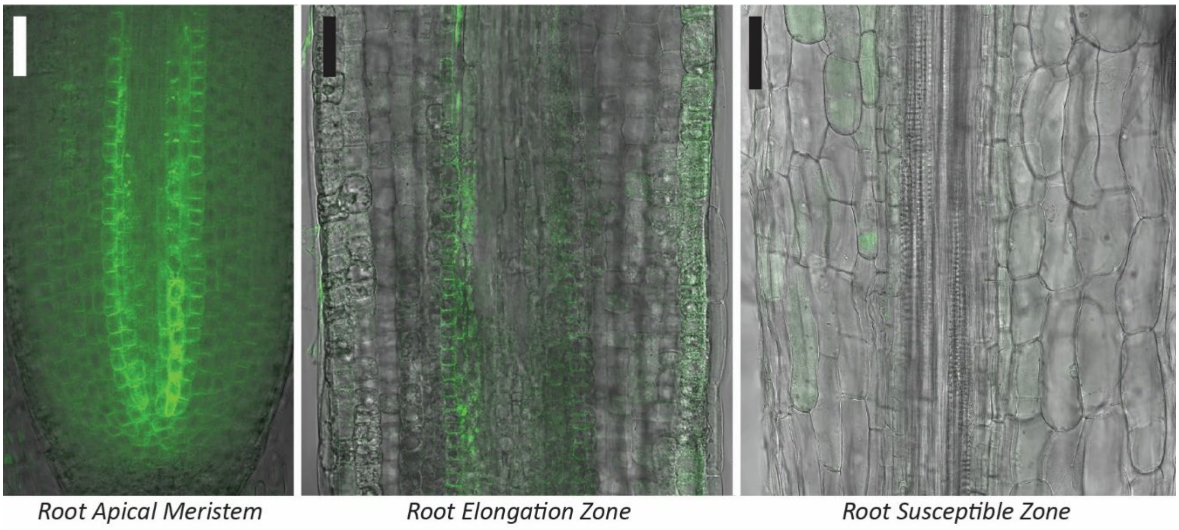

Supplementary Figure S12. Medicago PIN-FORMED10 (*Mt*PIN10) pattern in the Medicago root. *Mt*PIN10-GFP is accumulated in vasculature and detected in cortical cells of root meristem, it is basal localized there. *Mt*PIN10-GFP level decreased in elongation zone and became undetectable in root susceptible zone (scale bars 75 µm).

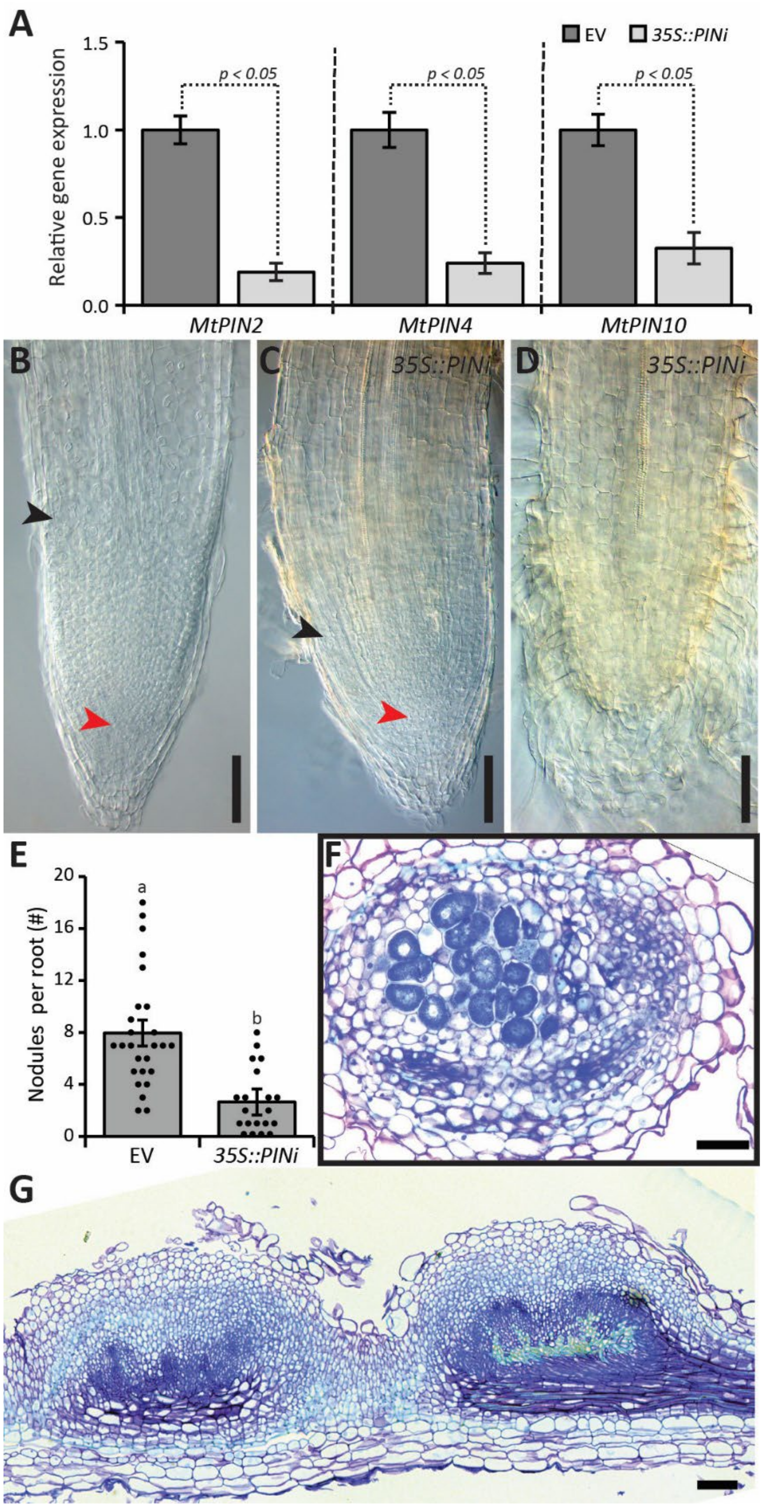

Supplementary Figure S13: Validation of RNA interference on Medicago *PIN-FORMED2* (*MtPIN2*), *MtPIN4* and *MtPIN10* in roots of composite Medicago A17 plants. **(A)** Relative expression of *MtPIN2*, *4* and *10* in transgenic *35S$_{pro}$::PIN2/4/10i* (*35S::PINi*) roots compared to *Empty Vector* (*EV*) control roots, bars represent averages, ± standard error, n=3. Statistical significance was determined based on Student's *t*-test, $p < 0.05$, as implemented in CFX Manager 3.0 software (Bio-Rad, Hercules, USA). **(B-D)** Representative Nomarski microscopy images of **(B)** an *EV* control root, **(C)** a *35S::PINi* root with a short root meristem and **(D)** a *35S::PINi* root without a root meristem. Arrowhead indicates quiescent center (red) and the end of the root meristem (black). Scale bars 75 µm **(E)** Number of nodules formed on *35S::PINi* transgenic roots compared to *EV* control roots, bars represent averages, ± standard error, dots show individual datapoints per transgenic root, different letters indicate significant differences with $p < 0.05$, according to ANOVA and Tukey post-hoc test, n>20. **(F-G)** Longitudinal plastic sections of a *35S::PINi* **(F)** nodule with a short and underdeveloped meristem and **(G)** root segment containing nodule-like bumps (both stained with toluidine blue; scale bars 50 µm).

SUPPLEMENTARY TABLES

Supplementary Table S1: Phenotype of *35S::PIN2/4/10 RNAi* transgenic roots

***RNAi* composite plants**	**Total roots analysed**	**Short roots (<3cm)**	**Longer roots (>3cm)**
EV	87	9	78
35S::PINi	89	62	27

Supplementary Table S2: List of primers

MtPINs protein GFP fusion constructs		
**Name**	**Sequence**	
MtPIN10-F1	GGGGACAACTTTGTATAGAAAAGTTGCGTGCTCGAATATTAGCCCAAC	
MtPIN10-R1	GGGGACTGCTTTTTTGTACAAACTTGCCATAGCTTCACCTTGAGATCCAG	
MtPIN10-F2	GGGGACAGCTTTCTTGTACAAAGTGGAGCCAACAAACATGCCAC	
MtPIN10-R2	GGGGACAACTTTGTATAATAAAGTTGGTGGCGGAATTCTTC	
eGFP-F	GGGGACAAGTTTGTACAAAAAAGCAGGCTCCCGGGGGTACCTGGTGAGCAAGGGCGAGGA	
eGFP-R	GGGGACCACTTTGTACAAGAAAGCTGGGTACTTGTACAGCTCGTCCATGC	
Primers for MtPINs promoter GFP fusion constructs		
**Name**	**Sequence**	
pMtPIN4-F2	TCGTGGTCAAGAACGTTCTC	
pMtPIN4-R	GTTTTTGAGTTGAGGTTTGAAGAAG	
pMtPIN10-F	TGCTCGAATATTAGCCCAAC	
pMtPIN10-R	TTTGTTTGGCTTATTGAAGTTTTG	
Primers for qRT-PCR		
**Name**	**Sequence**	**Gene ID Mt4 / r5.0**
YUC1qF	CTCAACTTGGGTTGGATCGT	Medtr3g109520 / MtrunA17_Chr3g0139441
YUC1qR	CCTTGAGCCAATAGGGAACA	
YUC2qF	GGGTGTGGAAATTCAGGTATGGAG	Medtr6g086870 / MtrunA17_Chr6g0485621
YUC2qR	AGGATGGATGAGCATTATGGTTGC	
YUC8qF	AGACTTTCTCTCACGCCGTTGC	Medtr7g099330 / MtrunA17_Chr7g0262591
YUC8qR	ACCAGATGGACCTGCACCTATG	
MtPIN2_01F	AAGAAAAGGGGAGGGAGGAG	Medtr4g127100 / MtrunA17_Chr4g0071571
MtPIN2_01R	CTTCTTTGTGCCAGCACTTG	
MtPIN4_01F	CTGTCAGATGCAGGACTTGG	Medtr6g069510 / MtrunA17_Chr6g0478431
MtPIN4_01R	CACACCCATTGAAAAAGCTG	
MtPIN10_02F	AAACACCGTTGCTTCTTTCG	Medtr7g089360 / MtrunA17_Chr7g0255941
MtPIN10_02R	CAATGTGCAACAGAACTCCC	
MtACTIN2_01F	TGGCATCACTCAGTACCTTTCAACAG	Medtr2g008050 / MtrunA17_Chr2g0278591
MtACTIN2_01R	ACCCAAAGCATCAAATAATAAGTCAACC	

RNAi constructs for Single MtPINi and MtYUCi	
**Name**	**Sequence**
MtPIN2-F	CACCATGATTACCGGTAAGGATATATAC
MtPIN2-R	CATTTCACCTATCTCTGCATCAG
MtPIN4-F	CACCCTACCATGTCATGACAGCAATG
MtPIN4i-R	TCCAATGGTGTTCTACCATCCAATG
MtPIN10i-F	CACCATACCATGTCCTCACAGCAG
MtPIN10i-R	ACCATCTTCACCAACTTCAGC
MtYUC1-F	GATCAGCTAGACGAAACATGCTACCACTC
MtYUC1-R	GCACTGTCTCATTAAACCGTG
MtYUC2-F	CACCATGGCACATGATCATCAAAAG
MtYUC2-R	AACATGCTACCACTCTTATAC
MtYUC8-F	CACCTCGTCTAGCTGATCACCAAGAC
MtYUC8-R	TCAAGTGAGAGTTCCATTCCAG
Double and triple MtPINi and MtYUCi	
**Name**	**Sequence**
MtPin4/10-R	AAGTCATGTCCACCAACCATCTTCACCAAC
MtPin10/4-F	GTTGGTGAAGATGGTTGGTGGACATGACTT
MtPin2/10-R	GTGAGGACATGGTATCATCTTCACCTATCT
MtPin10/2- F	AGATAGGTGAAGATGATACCATGTCCTCAC
MtYUC8/2-F	GAGTGGTAGCATGTTTCGTCTAGCTGATC
MtYUC2/8-R	GATCAGCTAGACGAAACATGCTACCACTC
MtYUC1/2-R	ATGATCATGTGCCATTCAAGTGAGAGTTCC
MtYUC2/1-F	TTAATGAGACAGTGCATGGCACATGATCAT

Supplementary Table S3: Gene probe sets used for ViewRNA *in situ* hybridization

**Gene name**	**Gene ID Mt4 / r5.0**	**Assay ID according to ThermoFisher Scientific**
MtNF-YA1	Medtr1g056530 / MtrunA17_Chr1g0177091	VPRWEK2 Type 6
MtYUC1	Medtr3g109520 / MtrunA17_Chr3g0139441	VPKA3EK Type 1
MtYUC2	Medtr6g086870 / MtrunA17_Chr6g0485621	VF1-6000670 Type 1
MtYUC8	Medtr7g099330 / MtrunA17_Chr7g0262591	VF1-6000768 Type 1
MtYUC9	Medtr1g069275 / MtrunA17_Chr1g0182991	VPCE3WM Type 1
MtPIN2	Medtr4g127100 / MtrunA17_Chr4g0071571	VF1-17677 Type 1
MtPIN4	Medtr6g069510 / MtrunA17_Chr6g0478431	VF1-20313 Type 1
MtPIN6	Medtr1g029190 / MtrunA17_Chr1g0159341	VPDJXGJ Type 1
MtPIN10	Medtr7g089360 / MtrunA17_Chr7g0255941	VF1-17681 Type 1
MtLAX2	Medtr7g067450 / MtrunA17_Chr7g0241841	VPZTD33 Type 1
